# *Dmd*^mdx^ mice have defective oligodendrogenesis, delayed myelin compaction and persistent hypomyelination

**DOI:** 10.1242/dmm.050115

**Published:** 2024-05-09

**Authors:** Andrea J. Arreguin, Zijian Shao, Holly Colognato

**Affiliations:** ^1^ Department of Pharmacological Sciences, Stony Brook University, Stony Brook, NY 11794, USA; ^2^ Medical Scientist Training Program (MSTP), Stony Brook University, Stony Brook, NY 11794-8651, USA

**Keywords:** Dystrophin, Dystrophin-glycoprotein complex, Duchenne muscular dystrophy, Oligodendrocyte, Oligodendrocyte progenitor cell, Myelination, Myelin compaction, Corpus callosum

## Abstract

Duchenne muscular dystrophy (DMD) is caused by mutations in the DMD gene, resulting in the loss of dystrophin, a large cytosolic protein that links the cytoskeleton to extracellular matrix receptors in skeletal muscle. Aside from progressive muscle damage, many patients with DMD also have neurological deficits of unknown etiology. To investigate potential mechanisms for DMD neurological deficits, we assessed postnatal oligodendrogenesis and myelination in the *Dmd^mdx^* mouse model. In the ventricular-subventricular zone (V-SVZ) stem cell niche, we found that oligodendrocyte progenitor cell (OPC) production was deficient, with reduced OPC densities and proliferation, despite a normal stem cell niche organization. In the *Dmd^mdx^* corpus callosum, a large white matter tract adjacent to the V-SVZ, we also observed reduced OPC proliferation and fewer oligodendrocytes. Transmission electron microscopy further revealed significantly thinner myelin, an increased number of abnormal myelin structures and delayed myelin compaction, with hypomyelination persisting into adulthood. Our findings reveal alterations in oligodendrocyte development and myelination that support the hypothesis that changes in diffusion tensor imaging seen in patients with DMD reflect developmental changes in myelin architecture.

## Teaser

**Summary:** The *Dmd^mdx^* mouse model for Duchenne muscular dystrophy mouse reveals suppressed production of oligodendrocyte progenitor cells from stem cell niches, altered dynamics of oligodendroglia in white matter and aberrant myelin ultrastructure.

## INTRODUCTION

Duchenne muscular dystrophy (DMD) is an X-linked recessive disorder that affects 1 in 5000 boys globally and is caused by mutations in the dystrophin (*DMD*) gene. The *DMD* gene is one of the largest genes in the human genome and its product, dystrophin, is controlled by multiple independent promoters that generate distinct isoforms (Ervasti et al., 1990). The function of full-length *dystrophin* gene product (Dp427), by far the best understood, is known to be a critical component of the dystrophin-glycoprotein complex (DGC), which provides a structural link between the extracellular matrix (ECM) and the cytoskeleton in contractile muscle cells (Constantin, 2014; Henry and Campbell, 1996). DMD typically presents between 2 and 5 years of age, with clinical findings including an abnormal gait, weakness and clumsiness. The loss of dystrophin uncouples the link between muscle ECM and cytoskeleton, leading to profound muscle degeneration and fibrosis, eventually leading to death due to cardiac and respiratory complications (Waldrop and Flanigan, 2019).

DMD patients are also at a higher risk of cognitive, learning and behavioral problems, yet the cellular and molecular basis of these changes remain unknown (Banihani et al., 2015; Dorman et al., 1988; Hinton et al., 2004; Ricotti et al., 2016). Nearly one-third of DMD patients present with cognitive impairment (IQ<70) (Chamova et al., 2013; Ricotti et al., 2016; Weerkamp et al., 2022). Language deficits include dyslexia (Billard et al., 1998; Astrea et al., 2015) and difficulty with working memory (Tyagi et al., 2020; Hinton et al., 2000, 2001). Patients with DMD are at a higher risk of developing attention deficit-hyperactivity disorder (Lee et al., 2022; Caspers Conway et al., 2015; Pane et al., 2012), autism spectrum disorder (Simone et al., 2021; Parisi et al., 2018; Fujino et al., 2018) and obsessive-compulsive disorder (Pascual-Morena et al., 2022; Lee et al., 2022; Thangarajh et al., 2019). Neuroimaging studies have revealed structural brain abnormalities, including smaller total brain and gray matter volumes (Doorenweerd et al., 2014; Lv et al., 2011; Mavrogeni et al., 2017), and decreased cerebral perfusion (Doorenweerd et al., 2017). Diffusion tensor imaging (DTI) studies on patients with DMD also suggest microstructural white matter changes (Fu et al., 2016; Preethish-Kumar et al., 2020; Doorenweerd et al., 2014), with reduced fractional anisotropy (FA) values, and increased mean and radial diffusivity in white matter tracts, including the corpus callosum, being reported (Fu et al., 2016; Preethish-Kumar et al., 2020; Doorenweerd et al., 2014). While reduced FA values and increased radial diffusivity can indicate myelin abnormalities, other factors, such as increased water content due to an impaired blood-brain barrier, can influence these DTI metrics, prohibiting their use as a conclusive determinant of myelin abnormalities. However, given that the timing and proper execution of myelination plays a critical role in neurological processes, such as learning and executive function, altered DTI metrics combined with the high incidence of cognitive and learning disabilities in patients with DMD suggest that the loss of dystrophin may impair developmental myelination.

Previous studies have revealed dystrophin expression in multiple areas of the brain, including neurons, astrocytes and oligodendrocytes (OLs) (Hoffman et al., 1988; Miike et al., 1989; Gorecki et al., 1991; Bies et al., 1992; Yoshioka et al., 1992; Lidov et al., 1993; Tokarz et al., 1998; Garcia-Cruz et al., 2023; Aranmolate et al., 2017), yet the cell and molecular mechanisms that contribute to neurological deficits in DMD remain largely unexplored. In addition, dystrophin expression in the brain appears to be highly complex, with different dystrophin gene products being regulated both spatially and temporally (Garcia-Cruz et al., 2023; Romo-Yanez et al., 2020). Of particular note, dystrophin expression and function has been identified in neuronal inhibitory synapses (Zarrouki et al., 2022; Vaillend and Chaussenot, 2017; Miranda et al., 2009) and in astrocytic terminal processes (also known as endfeet) of the blood-brain barrier (Nico et al., 2003, 2004), implicating these cells in DMD neuropathology. In addition, we have previously demonstrated that OLs and their progenitor cells express Dp427, Dp140 and Dp71, and that the *Dmd^mdx^* mouse, which selectively lacks Dp427, has less myelin basic protein (MBP) – a main component of myelin – in the developing postnatal brain, although MBP levels were normal in adult mice (Aranmolate et al., 2017). In this study, we therefore evaluated whether Dp427 influences developmental myelination, a process that occurs largely postnatally. We report perturbances in oligodendrogenesis in the ventricular-subventricular zone (V-SVZ) neural stem cell (NSC) niche and delayed accumulation of mature OLs in nearby white matter tracts, followed by pronounced alterations in myelin architecture that persist into adulthood. These findings provide new insight into the neurological changes in DMD and inform the pursuit of future therapeutic strategies for this devastating disease.

## RESULTS

### Dp427 regulates the emergence of oligodendrocyte progenitor cells in the V-SVZ

**Fig. 1.**
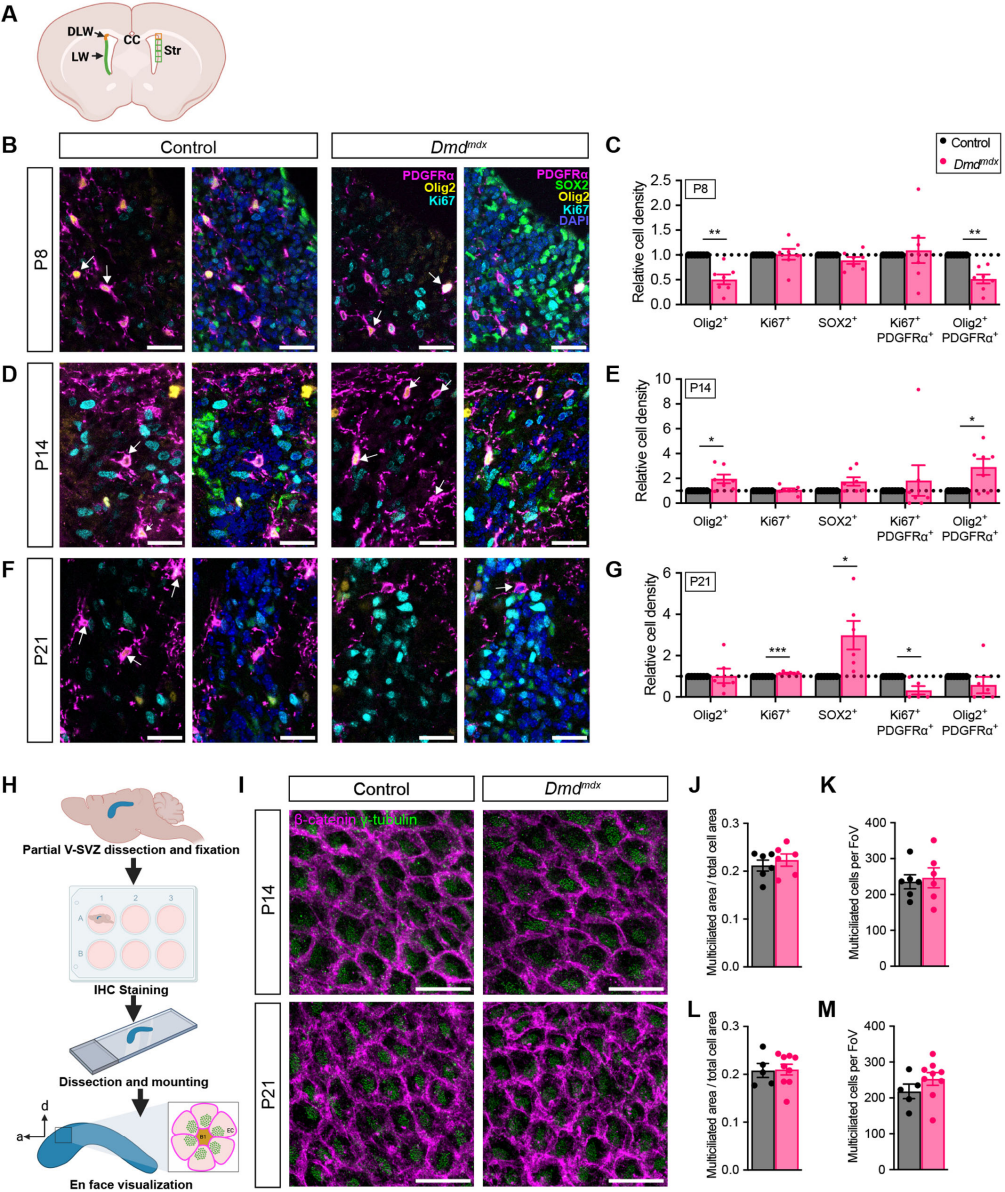
**The V-SVZ of *Dmd^mdx^* mice has fewer nascent oligodendrocyte progenitor cells (OPCs) than that of  control mice.** (A) Schematic of the V-SVZ, showing the dorso-lateral wedge (DLW, orange) and lateral wall (LW, green) regions. These regions were analyzed using immunohistochemistry of coronal brain sections from heterozygous (X^WT^X^MDX^) female (control) and *Dmd^mdx^* mice, to detect cells positive for Olig2, Ki67, Sox2, Ki67 and PDGFRα, or Olig2 and PDGFRα (Olig2^+^, Ki67^+^, Sox2^+^, Ki67^+^PDGFRα^+^ or Olig2^+^PDGFRα^+^, respectively) (see panels B, D and F). Boxed areas indicate the fields of view (FoV) for the DLW and LW. CC, corpus callosum; Str, striatum. (B,D,F) Representative immunohistochemistry images of the DLW region from control and *Dmd^mdx^* mice at postnatal days 8 (P8, B) P14 (D) or P21 (F) as indicated. Sections were immunostained to detect cells positive for Olig2, Ki67, Sox2, Ki67 and PDGFRα, or Olig2 and PDGFRα (Olig2^+^, Ki67^+^, Sox2^+^, Ki67^+^PDGFRα^+^ or Olig2^+^PDGFRα^+^, respectively). Boxes depict the fields of view (FoV) at 40× magnification. White arrows indicate Olig2^+^PDGFRα^+^ cells. (C,E,G) Bar graphs showing the relative cellular density of cells positive for proteins as indicated in the DLW of control and *Dmd^mdx^* mice at P8 (C), P14 (E) and P21 (G), as described for panels B, D and F. Multiple paired two-tailed *t*-test; *n*=7 (C,E), *n*=6 (G). *P<0.05, **P<0.01, ***P<0.001. (H) Schematic of whole-mount preparation of the LW V-SVZ, including immunohistochemistry (IHC) to detect γ-tubulin, to determine the basal body patch area and β-catenin in order to visualize apically located junctions between ependymal cells (ECs, pink outline), and to determine the total apical surface area. B1, type-B1 stem cell. (I) Representative images showing γ-tubulin-positive basal body patches (green) and β-catenin-positive ependymal cell apical cell junctions (magenta) in V-SVZ whole-mounts at P14 and P21. (J,K) Quantification of total area occupied by basal body patches (‘multiciliated area’) divided by total ependymal cell area in control and *Dmd^mdx^* mice at P14 (J) and p21 (L). Quantification of multiciliated cells per field of view (FoV) in control and *Dmd*^*mdx*^ mice at P14 (K) and p21 (M). For quantifications done at P14 (J,K), control and *Dmd*^*mdx*^ both *n*=6. For quantifications done at P21 (L,M), control: *n*=5 and *Dmd*^*mdx*^: *n*=9. At both P14 (J,K) and P21 (L,M), control versus *Dmd*^*mdx*^ comparisons were not significant (unpaired two-tailed *t*-test). All error bars denote ±s.e.m; all scale bars: 25 μm.

The loss of dystroglycan, a binding partner for dystrophin, has previously been shown to dysregulate the function of NSCs in the neonatal V-SVZ, resulting in both disturbed oligodendrogenesis and deficits in ependymal cell (EC) maturation that prevents the assembly of a normal NSC niche (McClenahan et al., 2016). We therefore examined the protein expression of Dp427 in the V-SVZ to determine its potential to influence the development of the postnatal V-SVZ. Using V-SVZ whole-protein lysates we found that Dp427 is present in the V-SVZ during all stages of postnatal development examined by us, until and including postnatal day 21 (P21) (Fig. S1A,B). To investigate whether Dp427 impacts oligodendrogenesis in the V-SVZ, we performed immunohistochemistry to analyze protein markers for NSCs and/or neural progenitor cells (NPCs) and oligodendrocyte progenitor cells (OPCs) in two gliogenic domains of the V-SVZ (Delgado et al., 2021) (Fig. 1A-G), the dorso-lateral wedge (DLW) and the lateral wall (LW). Both male (x^mdx^y) and female (x^mdx^x^mdx^) mutant mice, hereafter collectively referred to as *Dmd^mdx^* mice*,* were evaluated and compared to heterozygous female (X^WT^X^MDX^) littermates (controls). The DLW is a highly oligodendrogenic area that gives rise to the OLs and OPCs that populate the corpus callosum and, while the LW is also considered an oligodendrogenic domain, it is to a lesser extent under normal conditions (Marshall et al., 2003; Delgado et al., 2021; Chaker et al., 2016). Compared to wild-type littermates, loss of Dp427 in the *Dmd^mdx^* mice resulted in an increase in the NSC/NPC pool positive for SRY-box transcription factor 2 (SOX2^+^) by postnatal day 21 (P21) in the DLW, although this deficit was not present at earlier stages (P8 or P14; Fig. 1B-G). Interestingly, at P14, there were also more SOX2^+^ cells in the LW of *Dmd^mdx^* mice than by P21 (Fig. S1D-I). Next, we evaluated DWL and LW cells expressing the transcription factor Olig2, an OL lineage marker that is present in both OPCs and OLs (Sock and Wegner, 2021). *Dmd^mdx^* mice at P8 had fewer Olig2^+^ cells in the DLW than at P14 (Fig. 1B-E). At P21, the densities of Olig2^+^ cells in *Dmd^mdx^* DLW were comparable to those of control littermates (Fig. 1F,G). Next, we evaluated cells expressing platelet-derived growth factor receptor α (PDGFRα), an early marker of OPCs. We observed a similar trend to that seen when evaluating Olig2^+^ cells, such that the DLW of *Dmd^mdx^* mice comprised fewer Olig2^+^ PDGFRα^+^ OPCs at P8, yet more at P14 (Fig. 1B-E). To better appreciate expression of Olig2 and PDGFRa in individual cells,  Fig. S2 depicts single-channel views of the merged panels shown in Fig. 1B,D,F. The LW of *Dmd^mdx^* mouse V-SVZ has more Olig2^+^ PDGFRα^+^ OPCs at P14 (Fig. S1F,G) but fewer by P21 (Fig. S1H,I), similar to what was observed in the DLW. In contrast to what occurred in the DLW, however, we saw changes in Olig2^+^ PDGFRα^+^ cells within the LW without any change of Olig2 density in total. These findings reveal that loss of Dp427 leads to disturbances in the production of OPCs from the V-SVZ as well as an abnormally high number of SOX2^+^ cells by P21.

### Dp427 is not required for ependymal cell maturation and V-SVZ niche assembly

The V-SVZ stem cell niche develops perinatally, and its maturation and organization can affect NSC production of NPCs, including during oligodendrogenesis (Gonzalez-Perez and Alvarez-Buylla, 2011). To assess whether the content and architecture of the V-SVZ niche is normal in *Dmd^mdx^* mice, we used V-SVZ whole-mount immunohistochemisty, which provide the *en face* view that is needed to examine the organization of this NSC niche (Mirzadeh et al., 2010a) (Fig. 1H). Within the ventricular walls of the lateral ventricles, type-B NSCs are arranged as a pinwheel, where there is one or more centrally located type-B NSC surrounded by several ECs (Mirzadeh et al., 2008). ECs are specialized multiciliated cells that exhibit planar cell polarity, and the orientation of their motile cilia determine the movement of cerebral spinal fluid throughout the ventricles and allow for the vital movement of metabolites and trophic factors (Mirzadeh et al., 2010b). As ECs mature, they can be identified by their large apical surface and the presence of a large patch of γ-tubulin^+^ basal bodies (Spassky et al., 2005; Jacquet et al., 2009). And, since ECs develop in gradients, we measured solely in the dorsal mid-anterior region, which develops the most stereotypical pinwheel morphology (Mirzadeh et al., 2008) (Fig. 1H, bottom). Measuring the γ-tubulin^+^ basal body patch area compared to the apical surface area (where β-catenin immunoreactivity was used to delineate the cell-cell junctions), we did not observe any significant differences in the area coverage of basal body patches (relative to total cell area) between *Dmd^mdx^* and control littermates at P14 or P21 (Fig. 1I,J,L,M). Furthermore, we did not observe any differences in the number of multiciliated cells (a proxy for mature ECs) (Fig. 1K,N). Together these data indicated that the loss of Dp427 in the *Dmd^mdx^* mouse does not affect EC number or maturation.

### Dp427 regulates the proliferation of OPCs in the V-SVZ

To determine if changes in OPC cell densities in the V-SVZ could be due to altered proliferation, we next examined Ki67 immunoreactivity in PDGFRα^+^ OPCs. We observed a significant drop in the density of Ki67^+^ PDGFRα ^+^ at P21 in the *Dmd^mdx^* DLW, despite an increase in Ki67^+^ cells overall (Fig. 1G). One possibility was that proliferative stem and/or progenitors contribute to an overall – albeit small – increase in Ki67^+^ cells, despite a large drop in Ki67^+^PDGFRα^+^ cells. However, the mean change trend did not reach statistical significance (*P*=0.134; *n*=6). Likewise, the percentage of Sox2^+^ cells that were also Ki67^+^ was, on average, increased in *Dmd^mdx^* mice (2.26±0.87 fold), but also not statistically significant (*P*=0.170; *n*=6). In contrast to the DLW, we did not observe any differences in the density of Ki67^+^ PDGFRα^+^ or Ki67^+^ cells in the *Dmd^mdx^* LW (Fig. S1B-G). Overall, these data suggest that the output of V-SVZ oligodendroenic domains is altered in the *Dmd^mdx^* mouse, with the DLW being the one most significantly affected.

### *Dmd^mdx^* mice have fewer OPCs and OLs in the corpus callosum

**Fig. 2.**
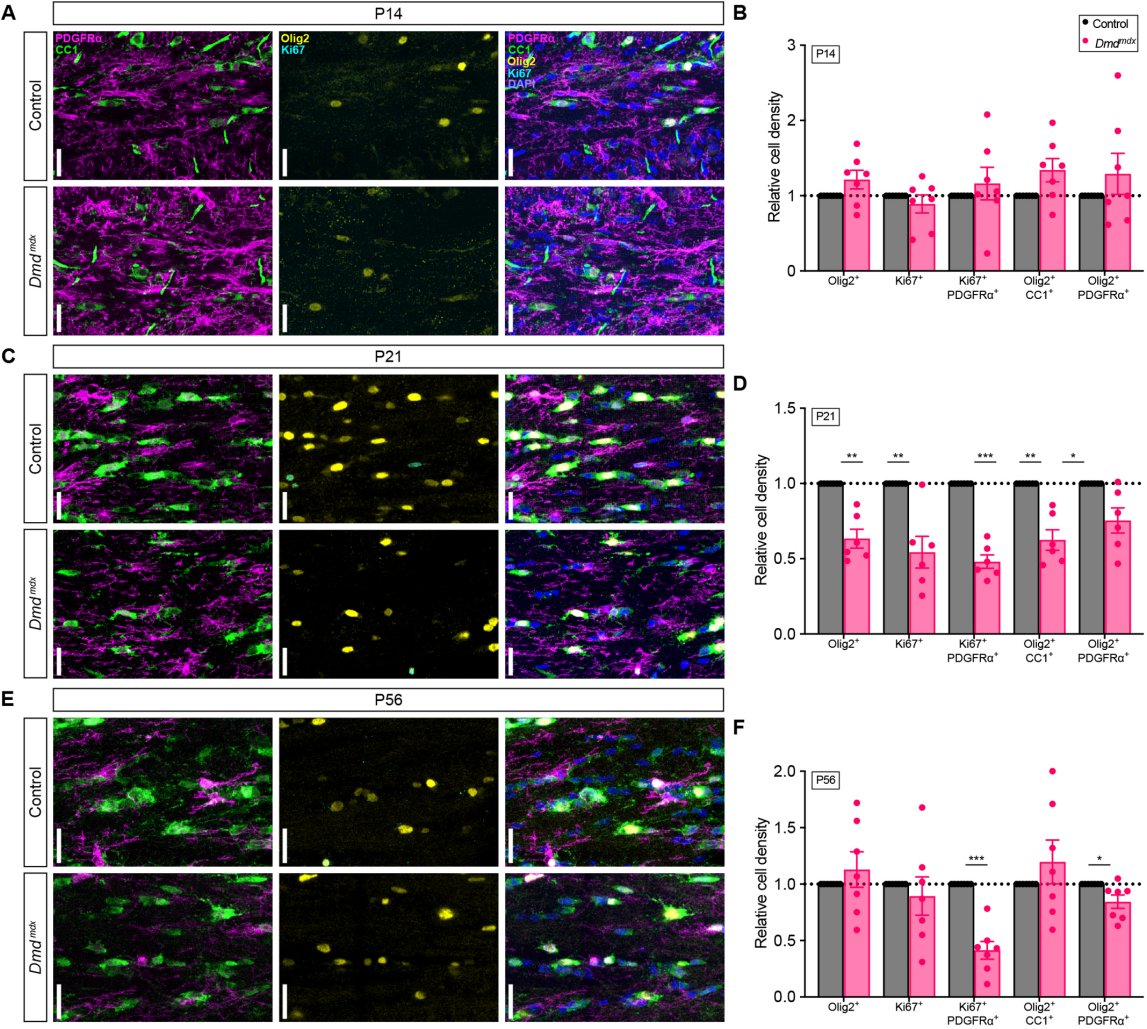
**Density of oligodendrocyte progenitor cells and oligodendrocytes is decreased in developing white matter of *Dmd^mdx^* mice.** (A,C,E) Representative images showing coronal sections of the corpus callosum from heterozygous (X^WT^X^MDX^) female (control) and *Dmd^mdx^* mice at P14 (A), P21 (C) and P56 (E) reveals cells immunoreactive for PDGFRα (magenta), CC1 (green), Olig2 (yellow) and Ki67 (cyan). (B,D,F) Bar graphs showing the relative cell density of cells positive for proteins as indicated (Olig2+, Ki67+, Sox2+, Ki67+PDGFRα+ or Olig2+PDGFRα+) in the corpus callosum of control and *Dmd^mdx^* mice at P14 (B), P21 (D) and P56 (F). *n*=7 (B,F), *n*=6 (D) Multiple paired two-tailed *t*-test; *P<0.05, **P<0.01, ***P<0.001. Error bars denote ±s.e.m in all graphs. All scale bars: 25μm.

We next sought to investigate whether the loss of OPCs and proliferating OPCs emerging from the V-SVZ impacted oligodendrogenesis in the corpus callosum. Using coronal floating sections, we examined Olig2^+^ PDGFRα^+^ OPCs, proliferating Ki67^+^ PDGFRα^+^ OPCs and Olig2^+^ CC1^+^ OLs (the latter detected by the monoclonal antibody 'anti-adenomatous polyposis coli clone CC1', also known as CC1, used to specifically label mature oligodendrocytes) in the corpus callosum during postnatal development (P8, P14 and P21), and at ∼2 months (P56). At P8 (pre-myelinating) and P14 (early myelination), *Dmd^mdx^* mice had similar densities of Olig2^+^ PDGFRα^+^ OPCs, Olig2^+^ CC1^+^ OLs and CC1^+^ OLs in their corpus callosum (Fig. 2A,B; Fig. S3A,B). At P21, however, *Dmd^mdx^* mice had fewer Olig2^+^ cells overall, and fewer OLs and OPCs in the corpus callosum (Fig. 2C,D). At P56, the density of OLs normalized to that in control littermates, but *Dmd^mdx^* mice still had fewer OPCs (Fig. 2E,F). Next, we wanted to determine if differences in cell densities could be explained by changes in cell death. Using the TUNEL assay, which identifies cells undergoing apoptosis, in combination with immunohistochemistry to detect oligodendroglial markers (Olig2, CC1, PDGFRα ) at P14, we examined the corpus callosum, as well as the DLW of the SVZ. Using the TUNEL readout, we did not observe any differences in cell death in the corpus callosum in *Dmd^mdx^* versus control mice; however, the *Dmd^mdx^* DLW in the SVZ had a small but statistically significant increase in cell death (Fig. S4). Since differences in In the density of OPCs could also be due to altered proliferation, we next examined OPC proliferation by assessing the density of Ki67^+^ PDGFRα^+^ cells in the corpus callosum. We found fewer proliferating Ki67^+^ PDGFRα^+^ OPCs and proliferating Ki67^+^ OPCs cells overall at P21 in *Dmd^mdx^* mice (Fig. 2A-D). To determine whether this phenotype persisted, we looked at *Dmd^mdx^* mice during young adulthood (P56), at which point we also observed fewer proliferating Ki67^+^ PDGFRα^+^ OPCs (Fig. 1E,F). These results indicate that loss of Dp427 prevents the development of normal OL densities in the corpus callosum at P21 but also that these cells catch up to those of control mice at ∼2 months of age. In contrast, *Dmd^mdx^* mice at P56 have still fewer OPCs and fewer proliferating OPCs.

### *Dmd^mdx^* mice have less MBP in the corpus callosum

**Fig. 3.**
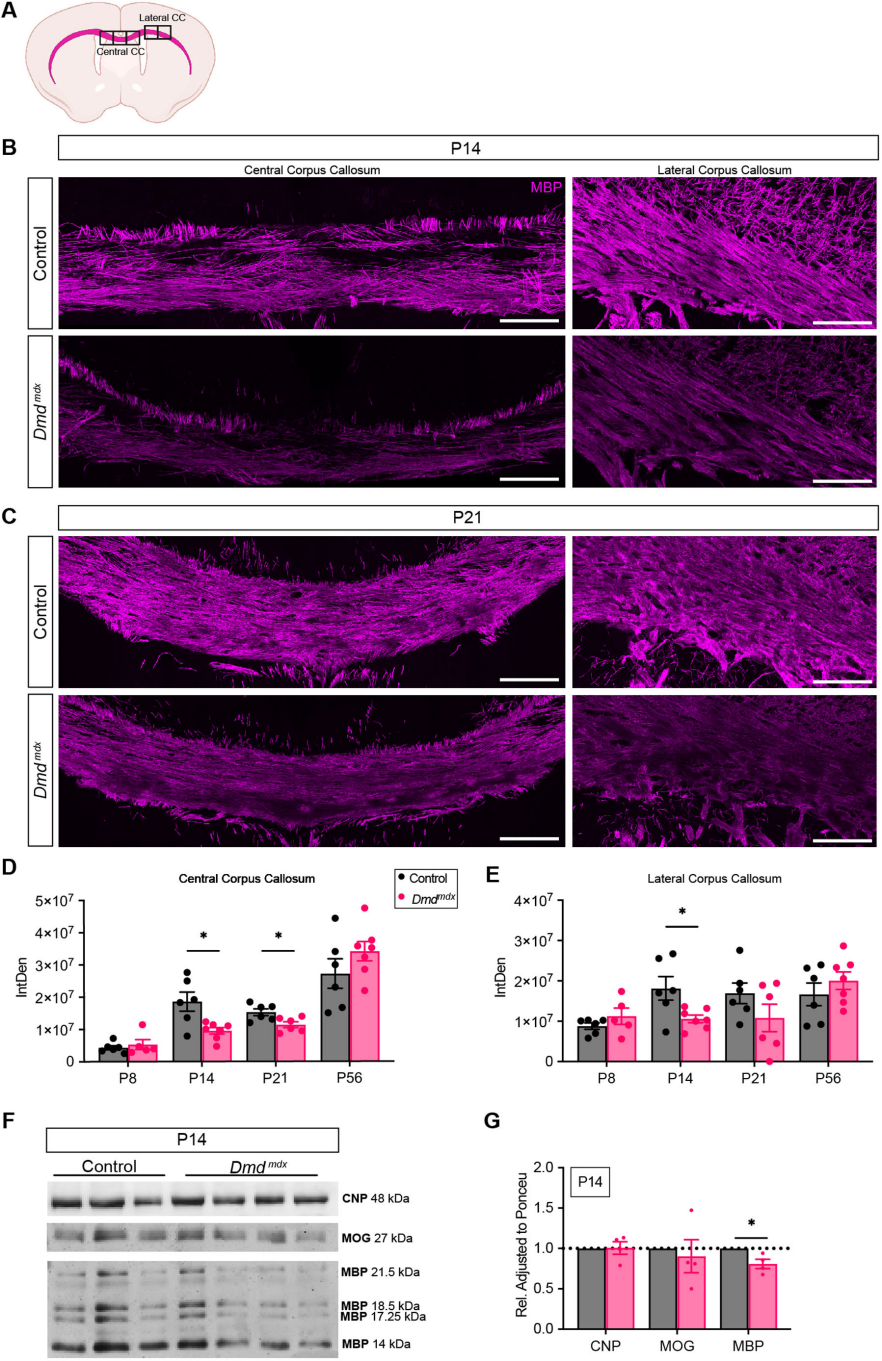
***Dmd^mdx^* mice have less MBP in the corpus callosum than control mice.** (A) Schematic of the brain, showing regions of the corpus callosum (magenta) that were analyzed for the intensity of the myelin basic protein (MBP). Boxed areas indicate the FoVs of the central and lateral corpus callosum (Central CC and Lateral CC, respectively). (B,C) Representative images of coronal sections from heterozygous (X^WT^X^MDX^) female (control) and *Dmd^mdx^* mice at P14 (B) or P21 (C), showing the central corpus callosum (left panels) and the lateral corpus callosum (right panels) following immunohistochemistry for MBP (red) visualized with anti-rat CY3. Scale bars: 100 μm. (D,E) Quantification of the integrated density (IntDen) of MBP in the central corpus callosum (D) or the lateral corpus callosum (E) of control and *Dmd^mdx^* mice at P8, P14, P21 and P56. *P<0.05, paired two-tailed *t*-test. P8: *n*=6 control, *n*=5 *Dmd^mdx^*; P14: *n*=6 control, *n*=7 *Dmd^mdx^*; P21: *n*=6 control and *n*=6 *Dmd^mdx^*; P56: *n*=6 control and *n*=7 *Dmd^mdx^*. Error bars denote ±s.e.m. (F) Western blots of lysates obtained from corpus callosum. The top membrane was probed for CNP at 48 kDa. The middle blot was probed for MOG at 27 kDa, and the bottom blot is from the same membrane as the middle, and it was probed for MBP (21.5, 18.5, 17.25 and 12 kDa). (G) Quantification of the western blot analysis of corpus callosum lysates probed for CNP, MOG and MBP (as shown in F). All protein band intensities were measured by using ImageJ and normalized to the intensity of total protein as determined by Ponceau staining in the entire lane. *P<0.05, paired two-tailed *t*-test. *n*=3 control and *n*=4 *Dmd^mdx^*. Error bars denote ±s.e.m.

Our current finding of fewer OLs provided a possible mechanism to explain the deficit in MBP levels in lysates obtained from whole cerebral cortices in our previous study (Aranmolate et al., 2017). To further examine MBP levels across the range of ages and obtain spatial information within the corpus callosum, we used immunohistochemistry on coronal floating sections to detect and quantify MBP immunoreactivity (Fig. 3). In *Dmd^mdx^* mice, we observed a decrease in intensity in the overall MBP immunoreactivity signal in both the central and lateral corpus callosum at P14, as well as a decrease in MBP signal in the central corpus callosum at P21 (Fig. 3B-E). However, by P56, the levels of MBP, as detected by using immunohistochemistry, were comparable to that of control (Fig. 3D,E). To further validate these results, we generated protein lysates from the corpus callosum and performed western blot analysis (Fig. 3F). *Dmd^mdx^* mice had less MBP protein at P14 (Fig. 3G), but at P21 and P57 levels were not significantly different than in control mice (Fig. S5). We also examined myelin proteins 2',3'-cyclic nucleotide 3' phosphodiesterase (CNP) and myelin oligodendrocyte glycoprotein (MOG) and did not observe any significant differences in their levels (Fig. 3G and Fig. S5).

### *Dmd^mdx^* mice have delayed myelin compaction

**Fig. 4.**
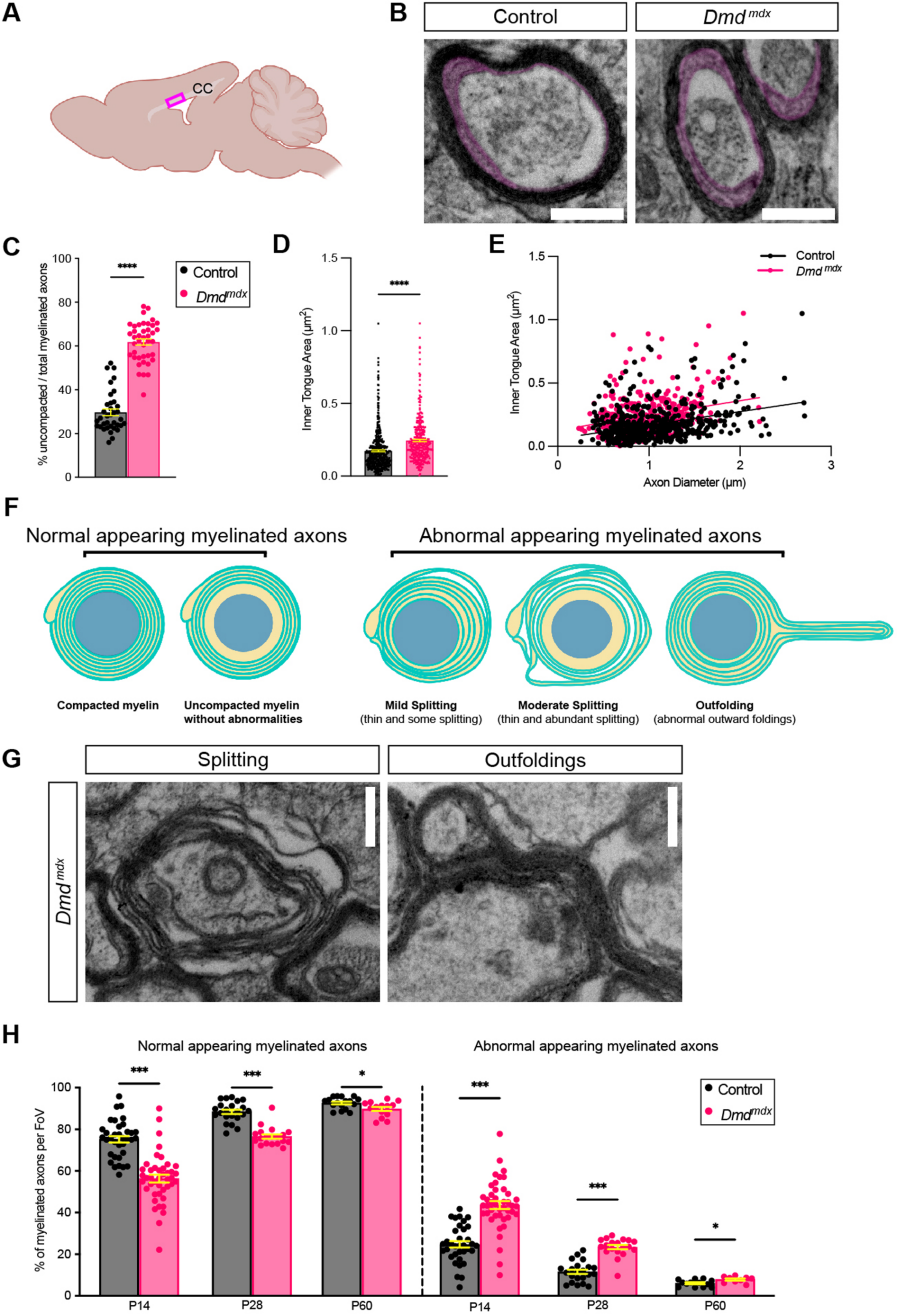
**During early myelination, the percentage of abnormal and uncompacted myelinated axons is increased in *Dmd^mdx^* mice.** (A) Schematic showing the coronal section of the corpus callosum (CC) dissected for transmission electron microscopy analysis. Boxed area shown indicates the region evaluated. (B) Electron micrographs of the OL cytosolic region, i.e. the inner tongue (magenta) obtained from heterozygous (X^WT^X^MDX^) female (control) (left) and *Dmd^mdx^* (right) mice. (C) Quantification of uncompacted and/or total myelinated axons (in %). ***P<0.001, unpaired two-tailed *t*-test. (D) Quantification of the inner tongue area (in µm^2^) measured from electron micrographs. A minimum of 150 axons per mouse was analyzed. ***P<0.001, unpaired two-tailed *t*-test. (E) Plotted is the inner tongue area (in μm^2^) over the axon diameter (in μm). ***P<0.001, simple linear regression of intercepts, not significant simple linear regression of slopes. (F) Schematics of myelinated axons that normal (left) or abnormal (right). Abnormal myelinated axons contained myelin outfoldings or various degrees of myelin splitting. (G) Representative electron micrographs of myelin splitting and outfoldings in *Dmd^mdx^* mice. (H) Quantification of myelinated axons (in %) per field of view (FoV) that appear normal (left) or abnormal (right) at P14, P28 or P60. Analyzed were a minimum of 1200 axons per genotype with ∼48 axons per FoV; 35 FoV for control and 42 FoV for *Dmd^mdx^* mice; *P<0.05, ***P<0.001, multiple unpaired two-tailed *t*-test, *P<0.05; ***P<0.001. All scale bars: 500 nm; all error bars in denote ±s.e.m.

The disturbances in OL maturation seen in *Dmd^mdx^* mice prompted us to determine if the myelination process itself was dysregulated. During myelination, mature OL processes wrap around axons many times, ultimately generating a mature multi-layered myelin sheath of stereotypical thickness relative to axon thickness. Aside from wrapping tightly around an axon segment, myelin undergoes compaction, a process by which the OL cytosolic region (also known as and hereafter referred to as ‘inner tongue’) that lies between the myelin layers and the axon cell surface largely disappears (Chang et al., 2016). Therefore, we used transmission electron microscopy to perform ultrastructural analysis of the myelinated axons of cross sections of the corpus callosum at P14, P28 and P60 (Fig. 4A). While a modest degree of uncompacted myelin is a normal feature of early myelination, we observed a significantly higher percentage of uncompacted myelinated axons within total myelinated axons in *Dmd^mdx^* mice at P14 than in control littermates (mean of 29.70±1.533% in control versus 61.82±1.420% in *Dmd^mdx^* mice) (Fig. 4B,C). At later time points, the percentage of uncompacted myelinated axons lessened in both control (18.38±2.21% at P28 and 8.14±0.58% at P60) and *Dmd^mdx^* mice (16.40±1.16% at P28 and 8.75±0.52% at P60) and was no longer significantly different (*P*=0.427 at P28 and *P*=0.440 at P60). To quantify the degree of compaction more precisely, we measured the inner tongue area as previously described (Meschkat et al., 2022; McNamara et al., 2022). We observed that *Dmd^mdx^* mice had a significantly larger inner tongue area (mean area 0.1735 μm±0.006 μm in control versus 0.2442 μm±0.008 μm in *Dmd^mdx^* mice) (Fig. 4D). Furthermore, we observed that inner tongue areas were larger in myelinated axons from *Dmd^mdx^* mice, regardless of axon diameter (Fig. 4E). These data suggest that myelin compaction is impaired during early myelination.

### Occurrence of abnormal myelin structures during early stages of myelination is more frequent in *Dmd^mdx^* mice

In addition to alterations in myelin compaction in *Dmd^mdx^* mice, we also noted a higher frequency of myelin structures that appeared abnormal, such as myelin splitting and outfoldings (Fig. 4F). For examples of myelinated axons with myelin splitting and myelin outfolding see Fig. 4G. Myelin splitting and outfoldings were seen throughout all ages (P14, P28 and P60) and were observed at a significantly higher percentage in *Dmd^mdx^* mice than in control littermates (Fig. 4H). This difference was the greatest at P14, where 43.64±1.514% of axons per field of view (FoV) in the *Dmd^mdx^* mice appeared abnormal, compared to only 24.77±1.827% in control littermates; and these findings were still substantial at P28 (23.42±1.016% in *Dmd^mdx^* versus 11.57±1.035% in control) (Fig. 4H). By P60, abnormal structures were rare but a small yet significant difference was still observed between genotypes (7.878±0.5027% in *Dmd^mdx^* versus 6.092±0.4497% in control) (Fig. 4H). Of note, the most abnormal structures present in either genotype were varying degrees of myelin splitting, whereas outfoldings were rare. The high percentage of these abnormal myelin structures in the *Dmd^mdx^* mouse, particularly during early myelination, indicates that Dp427 might be involved in early phases of myelin compaction.

### *Dmd^mdx^* mice have thinner myelin

**Fig. 5.**
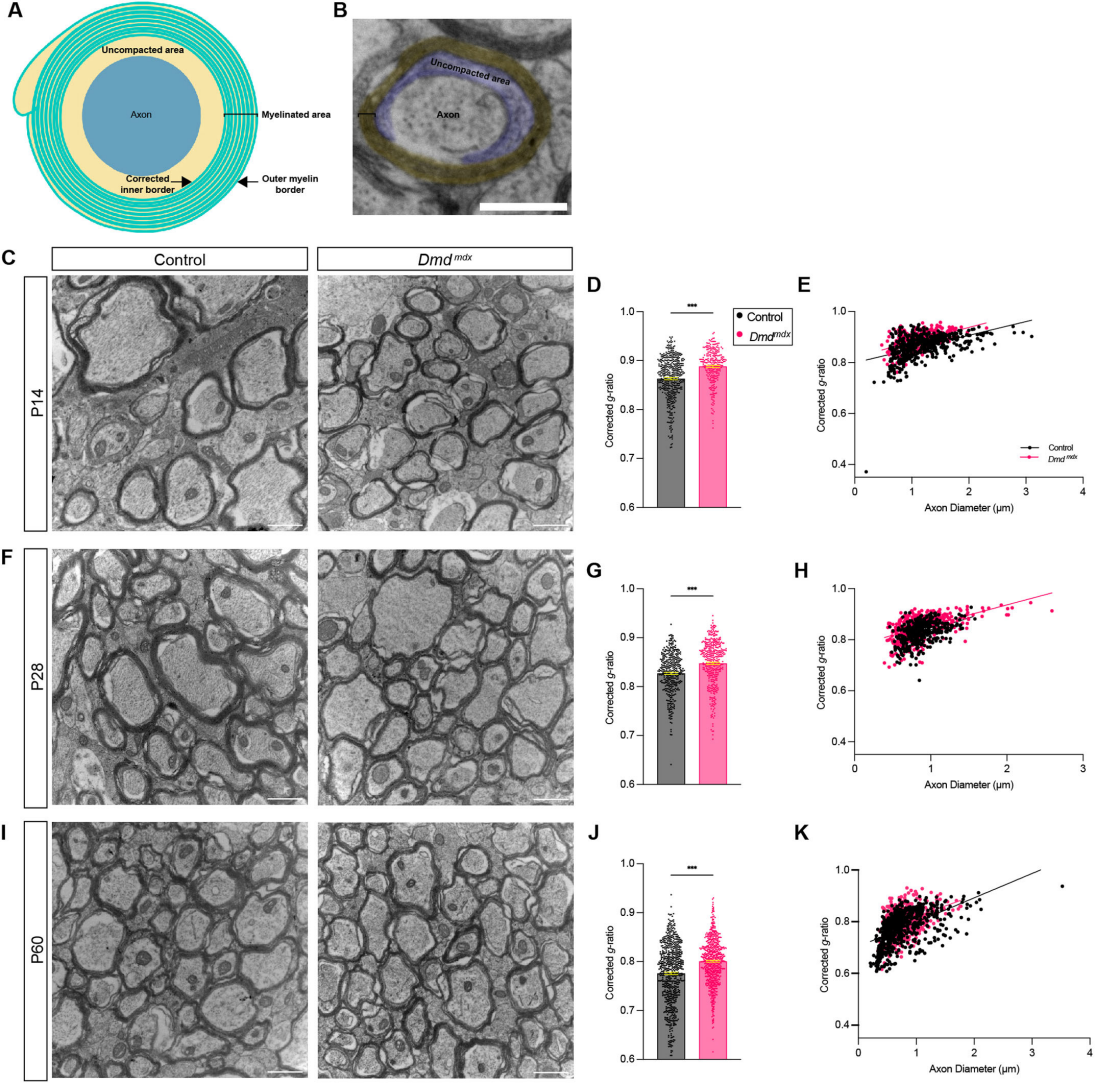
**The thickness of myelin layers in *Dmd^mdx^* mice is reduced at all ages assessed compared with that in control mice.** (A) Schematic of an uncompacted myelinated axon. The corrected inner border is indicated to illustrate how the myelin area is determined for the corrected *g*-ratio analysis. (B) Representative electron micrograph of a myelinated axon showing uncompacted (blue) and myelinated area (yellow). Scale bar: 1 µm. (C,F,I) Representative electron micrographs from the corpus callosum of heterozygous (X^WT^X^MDX^) female (control) and *Dmd^mdx^* mice at P14 (C), P28 (F) and P60 (I). Scale bars: 1 µm. (D,G,J) Bar graphs showing corrected *g*-ratio measurements from control and *Dmd^mdx^* mice at P14 (D), P28 (G) and P60 (J). ***P<0.001, unpaired two-tailed *t*-test. Error bars denote ±s.e.m. (E,H,K) Plotted is the corrected *g*-ratio over the axon diameter (in μm) of control and *Dmd^mdx^* mice at P14 (E), P28 (H) and P60 (K). ***P<0.001, simple linear regression of intercepts, not significant simple linear regression of slopes. A minimum of 150 axons per mouse was analyzed.

Next, we wanted to determine the thickness of the myelin by measuring the *g*-ratio, which determines the thickness of the compact myelin relative to the thickness of the axon. To adequately carry out this measurement, we calculated a ‘corrected *g*-ratio’ (Meschkat et al., 2022). As illustrated in Fig. 5A, the corrected *g*-ratio follows the inner myelin border rather than the axon border, to avoid incorrectly including the inner tongue area as myelin. We observed that, at all ages evaluated (P14, P28 and P60), *Dmd^mdx^* mice had significantly thinner myelin, i.e. a higher *g*-ratio, compared to control littermates. (Fig. 5C-K). We additionally binned *g*-ratios based on axon diameter and found that at P21, regardless of axon diameter, the myelin was thinner (Fig. S6). By P21 and P60, a pattern emerged in which axons with a diameter of ˂1.5 μm had significantly thinner myelin, whereas axons ˃1.5μm in diameter did not have a significant difference in myelin thickness (Fig. S6). We also examined the average diameters of myelinated axons in *Dmd^mdx^* mice and did not find any difference at P14, P28 or P60 (Fig. S7). Together these findings indicate that the process of myelin wrapping is abnormal in *Dmd^mdx^* mice, as both diminished and abnormal myelin wrapping were observed.

## DISCUSSION

**Fig. 6.**
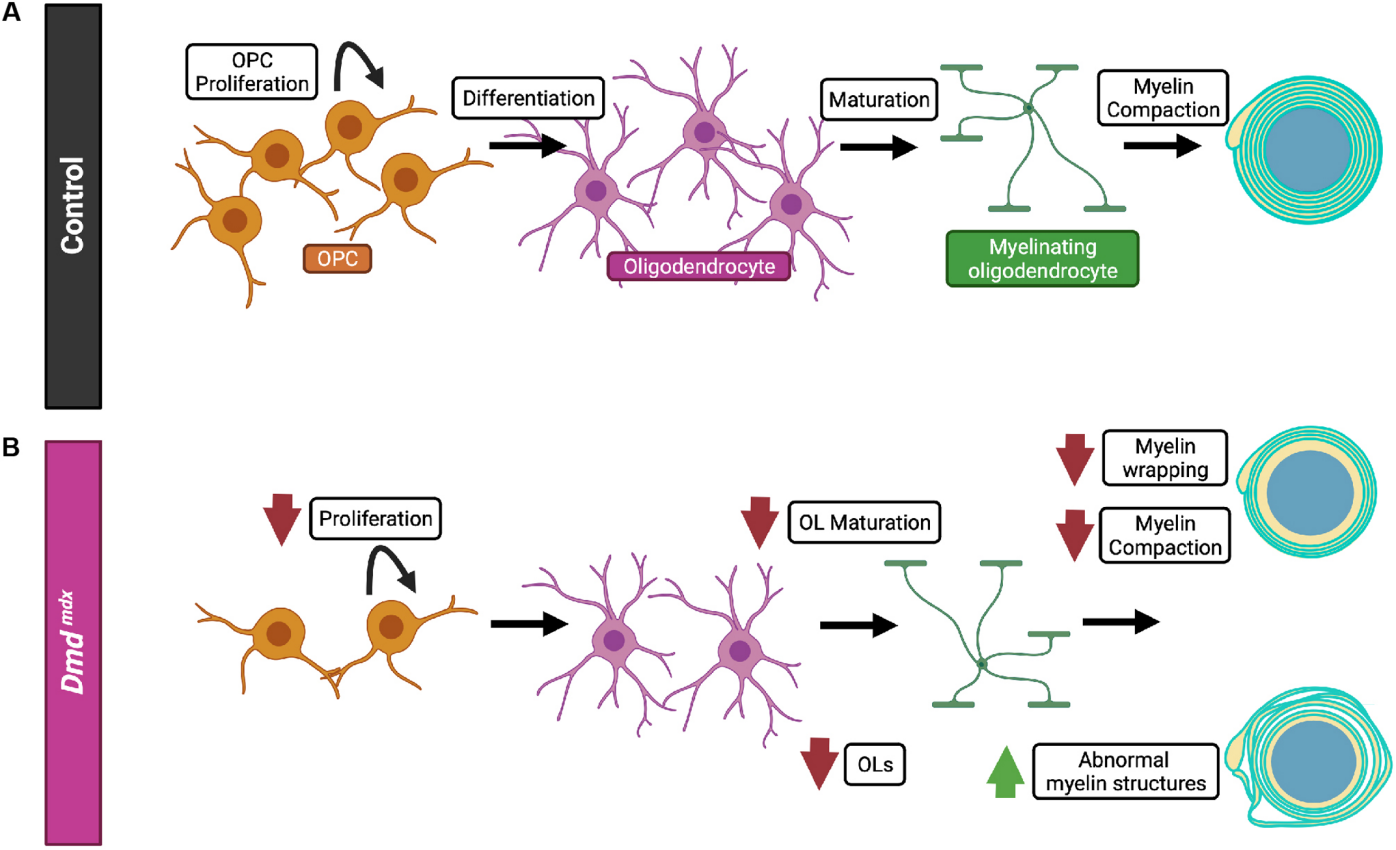
**Schematic of how loss of Dp427 disrupts oligodendrogenesis and myelination in the *Dmd^mdx^* mouse model.** (A) In heterozygous (X^WT^X^MDX^) female (control) mice normal postnatal oligodendrogenesis results in oligodendrocyte progenitor cells (OPCs) migrating out of the V-SVZ to populate the corpus callosum. OPCs can differentiate into oligodendrocytes (OLs) that mature, extend their processes, and begin to enwrap axons and to compact the myelin membrane. (B) In *Dmd^mdx^* mice fewer proliferating OPCs are present in the V-SVZ, their corpus callosum has fewer OPCs and mature OLs. Because of this impaired oligodendrogenesis, myelination in *Dmd^mdx^* mice is delayed, showing less myelin and less compact myelin. Furthermore, more myelin structures appear abnormal in *Dmd^mdx^* mice.

In this study, we reveal that the loss of Dp427 in *Dmd^mdx^* model mice lead to diminished production of OPCs in the V-SVZ NSC niche, despite having normal V-SVZ organization and normal degree of EC maturation. The corpus callosum of *Dmd^mdx^* mice had additional deficits, with decreases in both OPC proliferation and in mature OL densities. We also reveal that *Dmd^mdx^* mice had thinner myelin, an abundance of abnormal myelin structures – particularly in early stages of myelination – and a delay in myelin compaction. Together these abnormalities indicate that patients with DMD have pronounced differences in both the timing of myelination and in the resulting structure of myelin (see the schematic in Fig. 6). However, a limitation is that our current study compared mutant mice (a mixture of males and females) to mice that are female heterozygous (i.e. equivalent to carriers). However, using heterozygous mice as a control group was deemed essential as it allowed us to directly compare mutants to their littermates – an important and necessary consideration when evaluating early postnatal myelination – as the precise timing and extent of this developmental process can vary between litters (Campagnoni et al., 1987). Moreover, studies evaluating female mice that are heterozygous have shown that females with levels of dystrophin that are 50% below those of wild type still maintain a normal phenotype (van der Pijl et al., 2018). A second consideration is whether loss of Dp427 could influence the expression levels of other dystrophin isoforms. We, therefore, evaluated the levels of Dp140 and Dp71, both of which are expressed in OLs, and found no changes in the levels of either (Fig. S8).

The postnatal V-SVZ, adjacent to the lateral ventricles, is the largest NSC niche in the adult mammalian brain (Alvarez-Buylla and Garcia-Verdugo, 2002). The V-SVZ produces the vast majority of forebrain OPCs, and does so throughout life (Menn et al., 2006), with peak production during the postnatal period (Hill et al., 2014; Hamano et al., 1998). Loss of the dystrophin-binding partner dystroglycan in neonatal NSCs of the V-SVZ has previously been found to cause inappropriate neural progenitor development, i.e. increased oligodendrogenesis (McClenahan et al., 2016), leading us to hypothesize that *Dmd^mdx^* mice have similar abnormalities. However, when we examined NSC and OPC densities in distinct regions of the V-SVZ, i.e. the DLW and LW, we observed that the highly gliogenic DLW domain (Delgado et al., 2021) had more-pronounced differences, with *Dmd^mdx^* mice having significantly fewer Olig2^+^ PDGFRα^+^ OPCs at P8 compared to control littermates. However, the V-SVZ in *Dmd^mdx^* mice seemed to rebound from this deficit such that – by P14 – OPC densities were higher in the V-SVZ, and OPC densities were normalized by P21. It remains unclear whether these changes represent a change in the timing of oligodendrogenesis or, rather, an early failure that is compensated for by the presence of other dystrophins or other compensatory mechanisms. Interestingly, between P14 and P21 additional cellular changes occurred in the V-SVZ, as we observed increased proliferation (Ki67) and increased densities of SOX2^+^ cells. While SOX2^+^ cells represent a mixture of both NSCs and NPCs, SOX2 is an important transcription factor necessary for both proliferation and myelination of OPCs in the postnatal brain (Zhang et al., 2018). However, since we observed fewer proliferating OPCs during increased expression of SOX2, additional molecular changes are probably involved, which lead to OPC alterations. One possibility is that loss of dystrophin affects the number of astrocytes, which also express dystrophin (Nico et al., 2003; Garcia-Cruz et al., 2023). However, we did not observe changes in levels of GFAP, widely used a marker for astrocytes. Despite this, this current study cannot rule out potential changes in astrocyte function that impact on the cellular differentiation of OLs.

V-SVZ NSCs are organized in a pinwheel arrangement of one or more centrally located NSCs surrounded by several ECs (Mirzadeh et al., 2008), the support cells that influence NSC output (Del Bigio, 2010). The poor development and maturation of ECs following loss of dystroglycan in neonatal NSCs of the V-SVZ has previously been hypothesized to underlie at least some of the changes in oligodendrogenesis observed in dystroglycan-deficient brains (McClenahan et al., 2016). Similar to the loss of dystroglycan, loss of Dp427 leads to changes in oligodendrogenesis; however, we did not find any abnormalities in either development of ECs or in organization of V-SVZ niche pinwheels. Our data suggest that Dp427 is not required for V-SVZ niche assembly but, instead, regulates the production of OPCs (and their proliferation) via a distinct mechanism.

Once OPCs are made in the V-SVZ they migrate to adjacent structures, including the corpus callosum, where they can remain as resident OPCs or differentiate into OLs. We found *Dmd^mdx^* mice to have fewer OLs in the developing corpus callosum at P21, although OL densities caught up by adulthood. Despite the densities of OLs seeming normal during adulthood, OPC deficits and decreases in OPC proliferation emerged in the corpus callosum by P21, and persisted until the age of ∼2 months. Unlike the loss of dystroglycan, which resulted in increases of OPCs and OLs early on at P8, followed by normalization in OL lineage cells by P21 (McClenahan et al., 2016), the loss of Dp427 appeared to affect not only the differentiation of OPCs into OLs but also the pool of available OPCs. Moreover, the finding that the DLW of the V-SVZ NSC niche contained increased numbers of SOX2^+^ cells (NSC/NPCs) in conjunction with fewer OPCs, suggests that the transition from NSC/NPC to OPC in *Dmd^mdx^* is impaired.

There are several potential consequences of having a diminished OPC pool. OPCs are more heterogenous than previously thought and they have other roles in the CNS besides producing OLs (Hill et al., 2024). These roles include the sensing of potassium in the extracellular space (Maldonado et al., 2013), release of neuromodulatory factors (Sakry et al., 2015) and enwrapping, i.e. to surround axons in areas such as the V-SVZ (Delgado et al., 2021; Spitzer et al., 2019). Although these other roles are less understood, they demonstrate the capacity of OPCs to influence signaling and neuronal networks in the CNS. Furthermore, OPC proliferation in the corpus callosum typically decreases after peak myelination and plateaus until old age, such that OPC densities remain relatively stable (Spitzer et al., 2019). However, in *Dmd^mdx^* mice, we detected a reduction in the levels of OPC proliferation and the number of OPCs already at 2 months, despite finding no changes in the amount of cell death within the corpus callosum. This diminished OPC status might indicate an accelerated aging process in the OPC population of *Dmd^mdx^* mice.

Both human (Fu et al., 2016; Preethish-Kumar et al., 2020; Doorenweerd et al., 2014) and mouse studies (Xu et al., 2015) have similar imaging profiles in white matter tracts, displaying increased diffusivity and decreased FA, measurements that are consistent with hypo- or dysmyelination. Our previous study revealed that *Dmd^mdx^* mice may have a delay in myelination, as less MBP, a main component of myelin sheaths, was detected in lysates from the cerebral cortex at early stages of myelination (P14-P42) (Aranmolate et al., 2017). We sought to build upon these findings by examining MBP (and other myelin markers) specifically in the corpus callosum both during and after postnatal myelination. Similar to previous observations in the cerebral cortex, in the corpus callosum we observed an early deficit in MBP levels and normal levels in young adulthood, although CNP or MOG levels were not significantly different at any time point. We additionally examined MBP by using immunohistochemistry and found a similar pattern, i.e. early deficits followed by normalization at 2 months. These findings suggest that the loss of Dp427 only delays the expression of MBP, which could be due to the corresponding fewer OLs, albeit this deficit was not apparent until P21.

During myelin formation and compaction, an OL spirally enwraps myelin, and this process is dependent on MBP and CNP, which work to compact the myelin (driven by MBP) and form cytoplasmic channels (driven by CNP antagonizing MBP function) (Snaidero et al., 2014, 2017). Lack of MBP in the *shiverer* mouse prevents myelin compaction (Readhead et al., 1987; Rosenbluth, 1980), and ablation of MBP in mature OLs after a normal postnatal myelination results in a similar phenotype for newly added myelin (Meschkat et al., 2022). These studies demonstrate the importance of MBP in myelin compaction. In our current study we demonstrated reduced expression of MBP – but not CNP – at early postnatal days (P14 and P21), which may contribute to significantly attenuated myelin compaction. However, given that levels of CNP and MOG levels were not, and those of MBP only modestly affected, it seems likely that the deficits in myelin wrapping are due to mechanistic failures, such as faulty cell–cell or cytoskeletal interactions. Furthermore, we reported increased numbers of inappropriate myelin structures, particularly at P14, where we saw a significantly larger percentage of myelinated axons with abnormalities (myelin splitting), suggesting deficits in the process by which myelin ensheathment occurs.

Myelination is a highly dynamic process that requires initial actin assembly in the inner tongue (Zuchero et al., 2015; Nawaz et al., 2015) to drive ensheathment of the axon (Zuchero et al., 2015). Following ensheathment is membrane compaction, an MBP-dependent (Shine et al., 1992) process that drives the disassembly of the actin cytoskeleton in the outermost layers (abaxonal) and membrane extension of the inner tongue (adaxonal) (Snaidero et al., 2014; Zuchero et al., 2015). Correctly regulated actin dynamics are, therefore, critical to both myelin growth and compaction (Nawaz et al., 2015). It is possible that lack of Dp427, a protein that has known actin-binding sites, impairs OL actin dynamics at early stages of myelination. Since compaction eventually proceeded – as evidenced by thinner but seemingly properly compacted myelin at 2 months of age – this suggests that Dp427 either regulates the early stages of compaction and/or its timing, but that it is not required for compaction to proceed. It should be noted that at P14, i.e. when *Dmd^mdx^* compaction deficits are pronounced, *Dmd^mdx^* mice still have normal numbers of OLs in the corpus callosum. The precise molecular mechanism by which Dp427 is involved in myelin compaction remains to be further investigated.

Although *Dmd^mdx^* mice at all age groups investigated have thinner myelin layers, the continued decrease of *g*-ratio with age, might reflect a protracted delay in myelination rather than complete deficit. In other words, myelin continues to get thicker in both *Dmd^mdx^* and control littermates. This interpretation agrees with the observed levels of MBP, which normalize to those in control mice by the age of 2 months. A myelination delay can have profound consequences to neurodevelopment since myelin plays an important role in the formation and function of the neuronal circuitry. Indeed, changes in white matter in the corpus callosum have been implicated in conditions such as dyslexia (Sihvonen et al., 2021), ADHD (Cao et al., 2010; Qiu et al., 2011; Ameis et al., 2016) and autism spectrum disorder (Temur et al., 2019), which occur at higher incidences in patients with DMD. Related neurobehavioral deficits have been identified in DMD mouse models, including altered fear and anxiety responses, altered associative learning, and decreased social behaviors (Chaussenot et al., 2015; Miranda et al., 2015; Vaillend and Chaussenot, 2017; Zarrouki et al., 2022; Kreko-Pierce and Pugh, 2022; Wu et al., 2022). Anxiety, in particular, has a strong correlation with myelin deficits (Makinodan et al., 2009; Zhang et al., 2013; Serra-de-Oliveira et al., 2015; Xiu et al., 2017; Sen et al., 2019; Tomas-Roig et al., 2019; Cerina et al., 2020). It will be interesting to learn to what extent changes of myelin ultrastructure in DMD contribute to these and other behavioral deficits.

Overall, this current study suggests that white matter changes seen in patients with DMD can be due, in part, to deficits or delays in myelination and myelin compaction, with aberrant OPC pool expansion and proliferation a likely contributing factor. Also, given that dystrophin is known to mediate critical interactions with the actin cytoskeleton in skeletal muscle, an intriguing possibility that remains to be explored is whether oligodendroglial dystrophin regulates cytoskeletal dynamics during myelin ensheathment and compaction.

## MATERIALS AND METHODS

### Animals

All animal experiments were performed in accordance with the National Institutes of Health (NIH) Guide for the Care and Use of Laboratory Animals. A protocol for these studies was approved by the Stony Brook Institutional Animal Committee on Use and Care. Hemizygous males (X^MDX^; C57BL/10ScSn-Dmd- Mdx/J) and homozygous females (X^MDX^X^MDX^; C57BL/10ScSn-Dmd- Mdx/J) were purchased from The Jackson Laboratory (Bar Harbor, ME, USA). Two crosses were performed: 1) XY crossed with (X^MDX^X^MDX^) and, 2) X^MDX^Y crossed with XX^MDX^. Both male (X^MDX^Y) and female (X^MDX^X^MDX^) mutants were used and called *Dmd^mdx^*. In both types of cross, heterozygous females X^WT^X^MDX^ served as littermate controls in order to guard against litter-to-litter variability in developmental myelination. Genotyping was performed with DNA extracted from mouse tails using the previously described primer-competition PCR approach (Shin et al., 2011) and using the following primers: Common forward: 5′-GCGCGAAACTCATCAAATATGCGTGTTAGTGT-3′, Wild-type reverse: 5′-GATACGCTGCTTTAATGCCTTTAGTCACTCAGATAGTTGAAGCCATTTT-3′, Mutant reverse: 5′-CGGCCTGTCACTCAGATAGTTGAAGCCATTTTA-3′.

### Tissue preparation

Animals were anesthetized with isoflurane and then transcardially perfused with 0.1 M phosphate-buffered saline (PBS pH 7.2) to clear blood. Next, mice were perfused with 4% paraformaldehyde (PFA) diluted in PBS, then brains were removed and post-fixed overnight in 4% PFA in PBS. Mice at P14 and younger were immersion-fixed overnight in 4% PFA in PBS. Tissue was washed the following day in PBS, then immersed with 30% sucrose in PBS for 2-3 days at 4°C before freezing in Tissue-Tek OCT compound (Sakura Finetek) and stored at −80°C. Coronal free-floating sections (30 μm) were cut using a cryostat (Leica CM1900) at −16°C and stored in cryoprotectant solution three parts glycerol, three parts ethylene glycol, one part phosphate buffer (0.2 M) and three parts double distilled water (ddH_2_O) for storage at −20°C until immunohistochemistry.

### Immunohistochemistry

Tissue sections were blocked in 5% donkey serum with 0.1-0.5% Triton X-100 (Sigma) at room temperature for 1 h. Tissue was incubated with primary antibodies diluted in blocking solution at 4°C and placed on a gentle rocker overnight (between 12-16 h). After washing with PBS, sections were incubated with secondary antibodies diluted in blocking solution for 2 h at room temperature on a gentle rocker. Sections were washed in PBS and counterstained with 10 μg/ml 4′,6-diamidino-2-phenylindole, dihydrochloride (DAPI, Sigma) for 7 min before mounting with SlowFade Gold Antifade Mountant (Sigma) on glass slides with coverslips. The specificity of secondary antibodies was tested by performing immunohistochemistry without primary antibodies.

### Confocal fluorescence microscopy

Confocal images of floating coronal sections were acquired on a Leica SP8X confocal microscope (Leica Microsystems) using 40× and 63× objective operating on a Leica Application Suite X (LAS X) software. Images were processed and analyzed using ImageJ software, with the Cell Counter Plug-in being used for counting cells. All imaging parameters (laser, gain) were constant within each age group.

### Whole-mount immunofluorescence and analysis of the subventricular zone

The subventricular zone (SVZ) whole-mount immunofluorescence technique was adapted from a previously described method (Mirzadeh et al., 2010a), illustrated in Fig. 1H. Animals were anesthetized with isoflurane and transcardially perfused with PBS. Mice at P14 and P21 were perfused with 4% PFA in PBS as above and their brains partially dissected to allow for visualization of the lateral ventricular walls for better antibody penetration. Brains were post-fixed overnight in 4% PFA in PBS then washed with PBS the next day. Tissue was blocked in 5% donkey serum with 0.5% Triton X-100 at room temperature for 1 h on a gentle rocker. Tissue was incubated with primary antibodies diluted in blocking solution for 24 h at 4°C on a gentle rocker. Next, tissue was washed in PBS and incubated with secondary antibodies diluted in blocking solution for 24 h at 4°C on a gentle rocker. The tissue was washed in PBS and counterstained with DAPI for 7 min. Last, the lateral walls (LWs) were fully dissected and mounted with SlowFade Gold Antifade Mountant (Sigma) on glass slides with coverslips. Lateral walls were left at least 2 days to flatten before imaging on the confocal microscope at 63×. Multiciliated and total ependymal cell (EC) areas were calculated based on four to five fields of view (FoV) and between 138 and 351 cells per mouse, and analysis was performed using ImageJ. Each 63× FoV was 0.0149 mm^2^ and, therefore, between 0.0596 mm^2^ (four FoV) and 0.0745 mm^2^ (five FoV) were surveyed per mouse.

### Antibodies

The following primary antibodies were used at indicated dilutions: mouse anti-APC/CC1 [immunohistochemistry (IHC) 1:20; Millipore OP80], mouse anti-CNP [western blotting (WB) 1:1000; Millipore C5922], rabbit anti-Dystrophin (WB 1 µg/ml, Abcam AB15277), mouse anti-beta-dystroglycan (WB 1:250; Developmental Studies Hybridoma Bank), rat anti-Ki67 (IHC: 1:100, Invitrogen 4-5698-82), rat anti-MBP (IHC 1:200; WB 1:1000; BioRad AA82-87), rabbit anti-Histone H3 (1:750; Santa Cruz), rabbit anti-MOG (WB: 1:1000; Abcam ab108505), rabbit anti-GFAP (1:1000; DAKO), rabbit anti-Olig2 (IHC 1:100; Millipore AB9610), goat anti-PDGFRα , (IHC 1:100; R&D Systems AF1062), mouse anti-SOX2 (IHC 1:100; R&D Systems MAB2018), mouse (IgG1) anti-β-Catenin [immunofluorescence (IF) Wholemount 1:500; BD Transduction Labs 610153], rabbit anti-γ-Tubulin (IF Wholemount 1:500; Sigma T5192). Olig2 was visualized using anti-rabbit CY5, Ki67 was visualized using anti-rat Alexa-Fluor 488, Sox2 was visualized using anti-mouse CY3, and PDGFRα was visualized using antigoat Alexa-Fluor 680. The following secondary antibodies were used at indicated dilutions: anti-mouse IgG1 (IF Wholemount 1:500; Fisher A-21240), anti-rabbit IRDye 800 Donkey (1:2500; Licor AB_2715510), anti-mouse IRDye 800 Donkey (1:2500; Licor AB_621847), anti-rat (IRDye 800 Goat) (1:4000; Licor AB_2721932).

### Coronal section immunofluorescence analysis

V-SVZ cell counts were calculated with the observer unaware of the experimental conditions, and with four FoV (one DLW, three LWs) for P8 and five FoV (one DLW, four LWs) for P14 and P21 per mouse at 40×. Corpus callosum cell counts were calculated with the observer unaware of the experimental conditions, and with either four (P8, P14 and P21) or five (P56) FoV per mouse at 40×. Each 40× FoV was 0.03719 mm^2^ and, therefore, an area of 0.14876 mm^2^ was surveyed at P8 and an area of 0.18595 mm^2^ was surveyed at P14, P21 and P56. The integrated density of MPB on maximum projections was analyzed in the center of the corpus callosum (three FoV) and side (two FoV) at 40× using ImageJ for P8, P14 and P21, for a total analysis area of 0.18595 mm^2^. Imaging at ∼2 months of age (P56) was done at 10× magnification, with three FoV for the central corpus callosum and three FoV for the lateral corpus callosum, and a total area of 4.5444 mm^2^ being assessed.

### Electron microscopy

Mice were perfused with fixative (4% PFA, 2.5% glutaraldehyde in 0.1 M PBS). Brains were then incubated overnight at 4°C in the same fixative. Brains were rinsed in PBS and cut sagittally on a vibratome to obtain 50 μm thick sections. The following day sections were treated with 2% osmium tetroxide for 1 h, dehydrated through an ascending series of ethanol and embedded in Durcupan between sheets of Aclar for 48 h at 60°C. Ultrathin sections (60-90 nm) were cut on an ultramicrotome, acquired onto 300 mesh copper grids and stained with 2% aqueous uranyl acetate and 0.3% aqueous lead citrate. Images of thin sections were obtained using a FEI Tecnai BioTwinG2 transmission electron microscope.

### Myelin analysis

Normal, abnormal and uncompacted myelinated axons were quantified from multiple FoV, acquired from two control and two *Dmd^mdx^* mice at each age. The analyses for uncompacted myelin, inner tongue and *g*-ratio measurements were from a direct magnification of 18,500×, and included at least eleven FoV with a total area of at least 590 µm^2^ and at least 150 axons per mouse. For counting abnormal myelin structures, analyses were done from a direct magnification of 6800×, and included a minimum of 1200 axons per genotype and on average 48 axons per FoV; 35 FoV for control and 42 FoV for *Dmd^mdx^* mice. Axon diameter and myelin thickness were calculated from a measured area based on the assumption of circularity. See illustration in Fig. 5A for visualization on the exact areas used for area measurements. Axon diameter was calculated from the measured axonal area using the following Eqn (1):
(1)

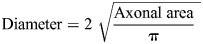
The inner tongue area was measured and the axonal area was subtracted from the inner tongue area as demonstrated in the following Eqn (2):
(2)

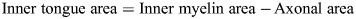
Since the inner tongue was enlarged, particularly at P14, a corrected *g*-ratio approach was used to analyze myelin thickness. For the corrected *g*-ratio the following Eqn (3) was used:
(3)

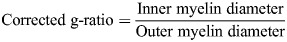


### Protein lysates

Tissue from each corpus callosum and V-SVZ were harvested and lysed with 20 mM Tris (pH 7.4), 1% sodium dodecyl sulfate and 1:50 dilution of protease and phosphatase inhibitor cocktails (Set II, Calibiochem) at 95°C for 15 min, with trituration at 10 min. Lysates were then centrifuged at 16,110 ***g*** for 10 min on a countertop centrifuge to remove insoluble material. Protein concentration was determined using a Bradford protein assay (Bio-Rad). Tissue lysates were prepared for SDS-PAGE with TruPAGE LDS sample buffer (Sigma) with 12% β-mercaptoethanol (Sigma).

### Western blotting

Protein (8 µg for analysis of mice at P14, and 4 µg for analysis of mice at P21 and P57) for corpus callosum lysates was separated by sodium dodecyl sulfate polyacrylamide gel electrophoresis (SDS-PAGE) on 18% acrylamide gels (1 mm thick) and transferred onto a 0.45 μm nitrocellulose membrane for 90 min at 100 V at 4°C. Protein (30µg) for V-SVZ lysates was separated by SDS-PAGE on a 7.5% acrylamide gel. Membranes were incubated 5 min with ponceau stain (0.1% v/w 5% acetic acid), washed 2×5 min in ddH_2_O and scanned to measure total protein per lane. Next, membranes were blocked with 4% bovine serum albumin (BSA) in Tris-buffered saline (TBS) with 0.1%Tween-20 (TBS-T) for 1 h at room temperature and then incubated overnight with primary antibodies diluted in blocking solution. The next day, membranes were washed with TBS-T 3×5 min and incubated with secondary antibody for 1 h at room temperature. Membranes were washed with TBS-T 3×5 min and 1× ddH2O 5 min prior to detection. Membranes with corpus callosum lysates were scanned on the LI-COR Odyssey. For V-SVZ lysates, membrane was incubated for 1 min at room temperature using Pierce ECL Western Blotting Substrate (Fisher Scientific). Substrate was removed and placed in a plastic sheet protector and covered in a film cassette. In the dark room, the membrane was exposed to autoradiography film. Protein band intensity was measured on ImageJ and normalized to total protein (overall ponceau staining within the entire lane).

### Statistical analysis

Statistical analyses were performed using Microsoft Excel and GraphPad Prism9. *P*-values, determined using unpaired or paired two-tailed tests as indicated, are: *P<0.05, ***P<0.01, ****P<0.001 and *****P<0.0001. Throughout, error bars represent the ±standard error of the mean (±s.e.m.). For slopes comparing *g*-ratio and inner tongue area to axon diameter, we used simple linear regression analysis.

## Supplementary Material

10.1242/dmm.050115_sup1 Supplementary information
