# Supplementary material for: Dmdmdx mice have defective oligodendrogenesis, delayed myelin compaction and persistent hypomyelination
Source: Dis Model Mech. 2024 May 9;17(4):dmm050115. doi: 10.1242/dmm.050115 (PMC11095635; doi:10.1242/dmm.050115)
Supplement: Supplementary information [file dmm-17-050115-s1.pdf]

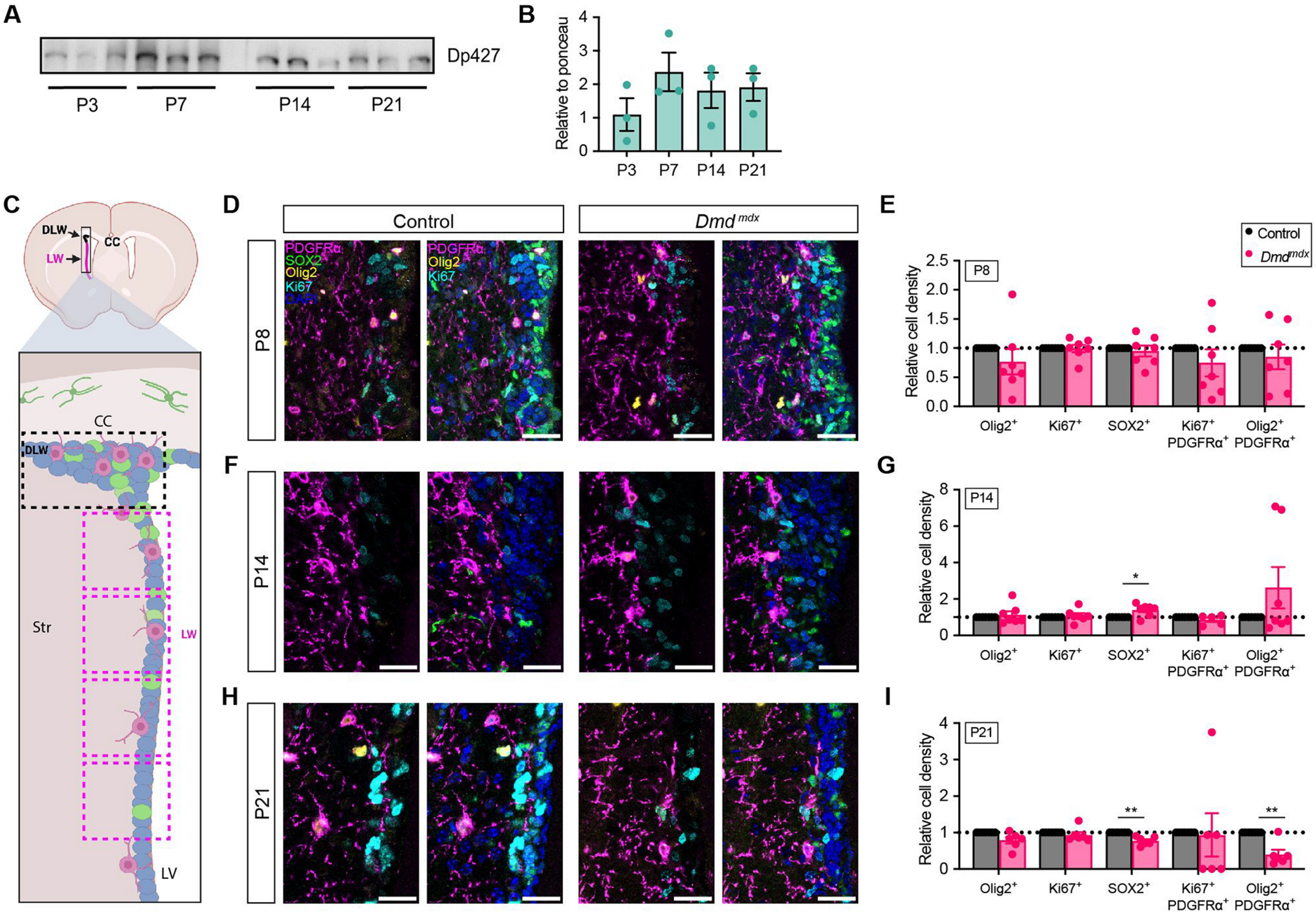

Fig. S1. Fewer oligodendrocyte progenitor cells in the lateral wall of the Dmd^{mdx} V-SVZ

(A) Dp427 protein expression in the V-SVZ of C57BL6 mice determined by western blotting. (B) Western blotting quantification normalized to total protein by ponceau staining. n=3 littermates per age. (C) Schematic of the dorsolateral wedge (DLW; black) and lateral wall (LW; magenta) regions of the V-SVZ that were analyzed using immunohistochemistry of coronal sections to detect Olig2$^+$, Ki67$^+$, Sox2$^+$, Ki67$^+$PDGFRα$^+$, and Olig2$^+$PDGFRα$^+$ cells. Boxes depict the fields of view at 40X magnification. (D) Representative images in the LW at P8 from control and Dmd^{mdx} mice. (E) Relative cell densities of indicated cell types in the LW control and Dmd^{mdx} mice at P8. Not significant, multiple paired two-tailed t-test; n=7. (F) Representative images from the LW from control and Dmd^{mdx} mice at P14. (G) Relative cell densities of indicated cell types in the DLW control and Dmd^{mdx} mice at P14. Multiple paired two-tailed t-test, n=7. *P*-values shown on graph. (H) Representative images in the LW at P21 from control and Dmd^{mdx} mice. (I) Relative cell densities of indicated cell types in the LW control and Dmd^{mdx} mice at P21. Multiple paired two-tailed t-test, n=6. *P*-values shown on graph. Error bars denote SEM for all graphs. * $P<0.05$, ** $P<0.01$. Scale bar 25μm for all images. *Abbreviations: corpus callosum (CC); striatum (Str); lateral ventricle (LV).*

Disease Models & Mechanisms • Supplementary information

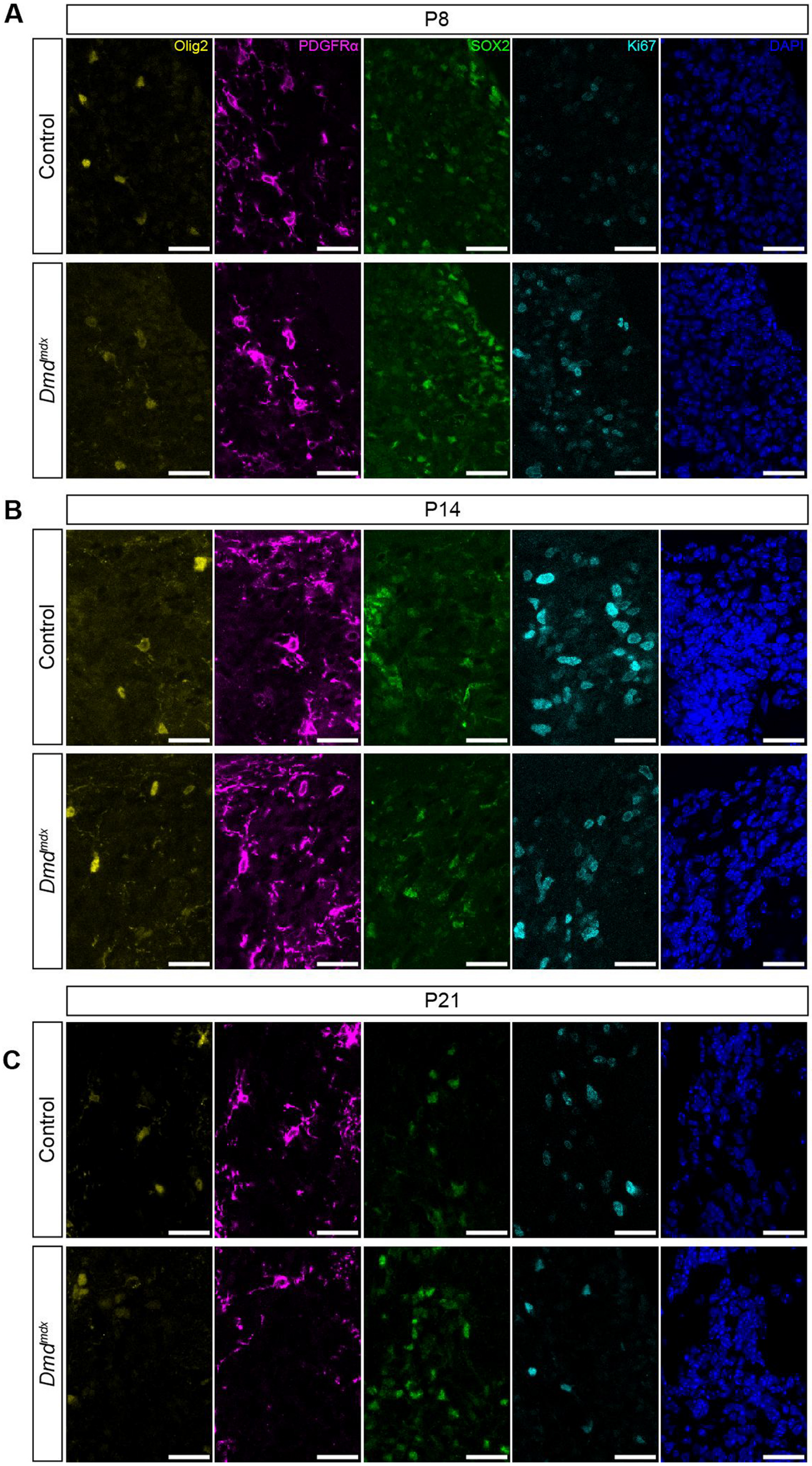

Disease Models & Mechanisms • Supplementary information

Fig. S2. Single channel views of oligodendrocyte progenitor cells in the *Dmd^mdx^* V-SVZ. Single channel views to accompany analysis of *Dmd^mdx^* V-SVZ oligodendrocyte progenitor cells in Figure 1B, D, and F are shown. Olig2 was visualized using anti-rabbit CY5, Ki67 was visualized using anti-rat Alexa488, Sox2 was visualized using anti-mouse CY3, and PDGFRα was visualized using anti-goat Alexa680. (A) Representative images in the DLW at P8 from control and *Dmd^mdx^* mice. (B) Representative images from the DLW from control and *Dmd^mdx^* mice at P14. (C) Representative images in the DLW at P21 from control and *Dmd^mdx^* mice. Scale bar 25µm for all images.

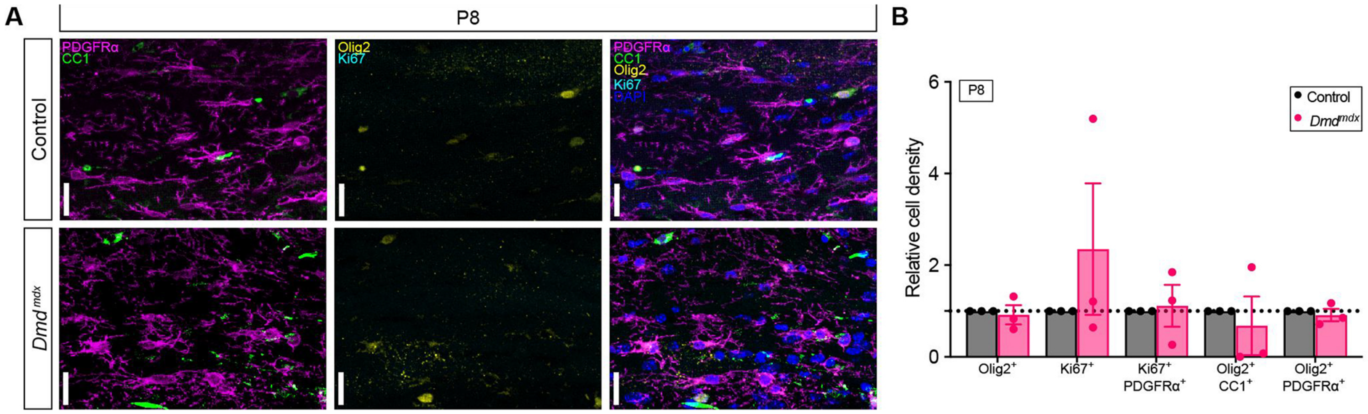

Fig. S3. Detection of oligodendrocyte progenitor cells and oligodendrocytes in the corpus callosum at P8

(A) Representative images from coronal sections of the corpus callosum from P8 control and *Dmd^mdx^* mice reveals cells immunoreactive for PDGFRα (magenta), CC1 (green), Olig2 (yellow), and Ki67 (cyan). (B) Relative cell densities of indicated cell types in the corpus callosum from P14 control and *Dmd^mdx^* mice. n=3, paired two-tailed t-test, not significant.

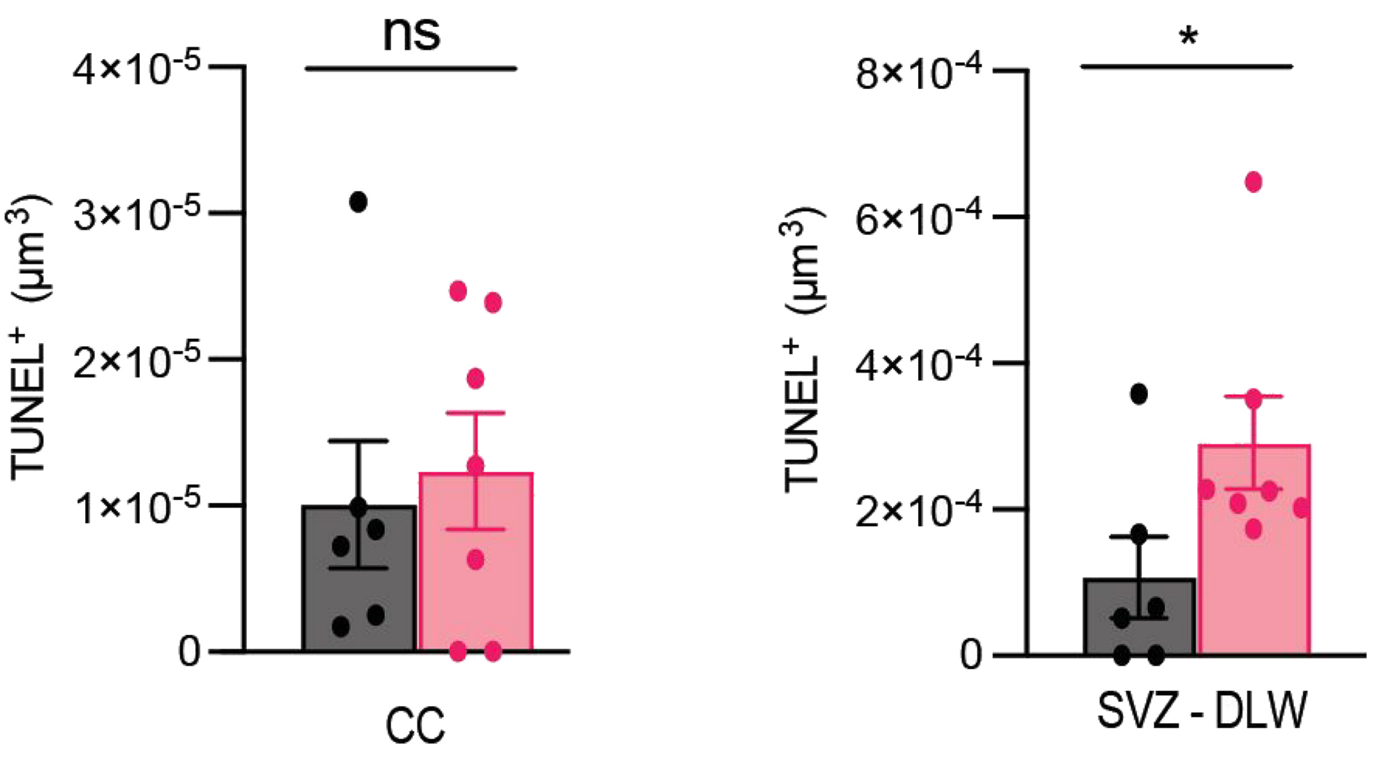

Fig. S4. Detection of TUNEL+ cells in the corpus callosum and V-SVZ of *Dmd^mdx^* mice. (A) TUNEL+ cells in the corpus callosum (CC) in control (grey bars) and *Dmd^mdx^* (pink bars) mice. (B) TUNEL+ cells in the dorso-lateral wedge (DLW) of the subventricular zone (SVZ) neural stem cell niche in control (grey bars) and *Dmd^mdx^* (pink bars) mice. n=6; Mann-Whitney rank comparison, *p<0.05 or not significant (ns)

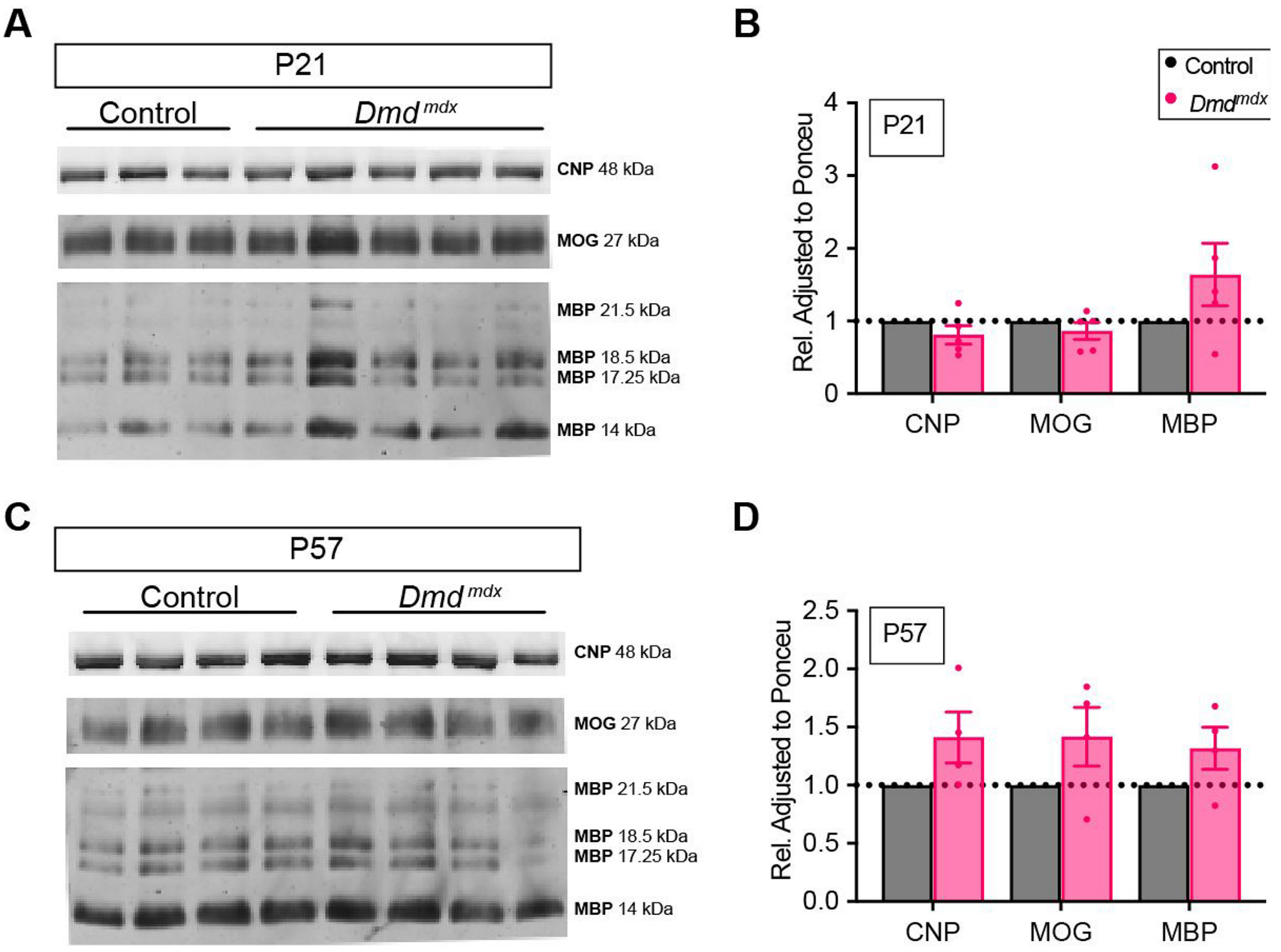

Fig. S5. Western blots to detect CNP, MOG, and MBP levels in the corpus callosum of P21 and P56 *Dmd*[*mdx*] mice.

Western blots of corpus callosum lysates from P21 (A) and (C) P57. The top membrane was probed for CNP (48 kDa). The middle blot was probed for MOG (27kDa) and the bottom blot is from the same membrane as the middle, and it was probed for MBP (21.5, 18.5, 17.25, and 12 kDa). (B) Quantification of western blot analysis of corpus callosum lysates from P21 probed for CNP, MOG and MBP. (D) Quantification of western blot analysis of corpus callosum lysates from 2 months (P57) probed for CNP, MOG and MBP. Not significant, unpaired two-tailed t-test. n= 3 control and n=5 *Dmd*[*mdx*] (P21), and n= 4 control and n=4 *Dmd*[*mdx*] (P57). Error bars denote SEM.

Disease Models & Mechanisms • Supplementary information

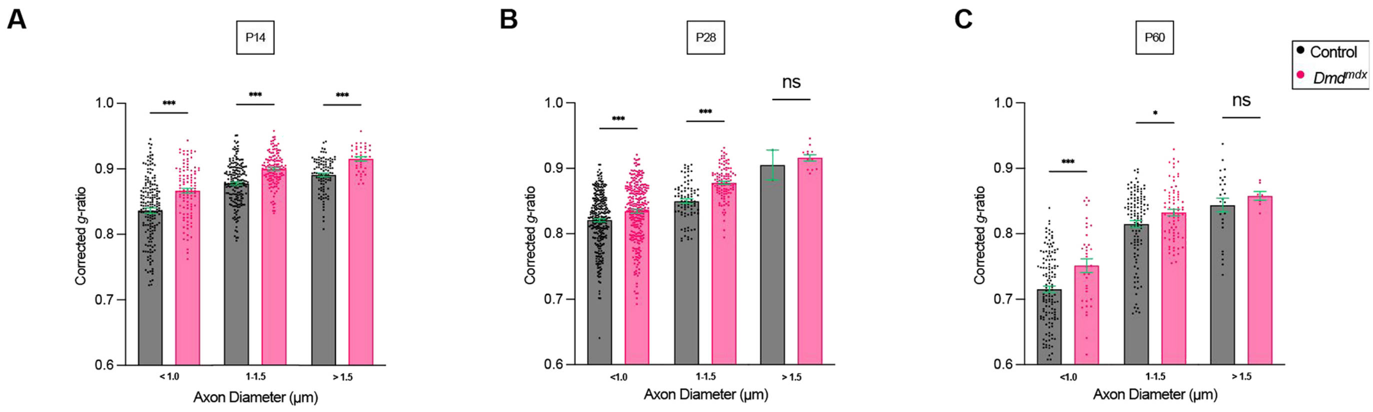

Fig. S6. Smaller diameter axons (<1.5 μm) have thinner myelin at P14, P28 and P60

(A) Corrected *g*-ratio measurements from P14 control and *Dmd*^{mdx} mice for axons <1.0μm, 1-1.5μm and >1.5μm. *** *P*<0.001, unpaired two-tailed t-test. (B) Corrected *g*-ratio measurements from P28 control and *Dmd*^{mdx} mice for axons <1.0μm, 1-1.5μm and >1.5μm. ** *P*<0.01, *** *P*<0.001, unpaired two-tailed t-test. (C) Corrected *g*-ratio measurements from P60 control and *Dmd*^{mdx} mice for axons <1.0μm, 1-1.5μm and >1.5μm. * *P*<0.05, ** *P*<0.01 unpaired two-tailed t-test. n= 150 axons per mouse. Error bars denote SEM.

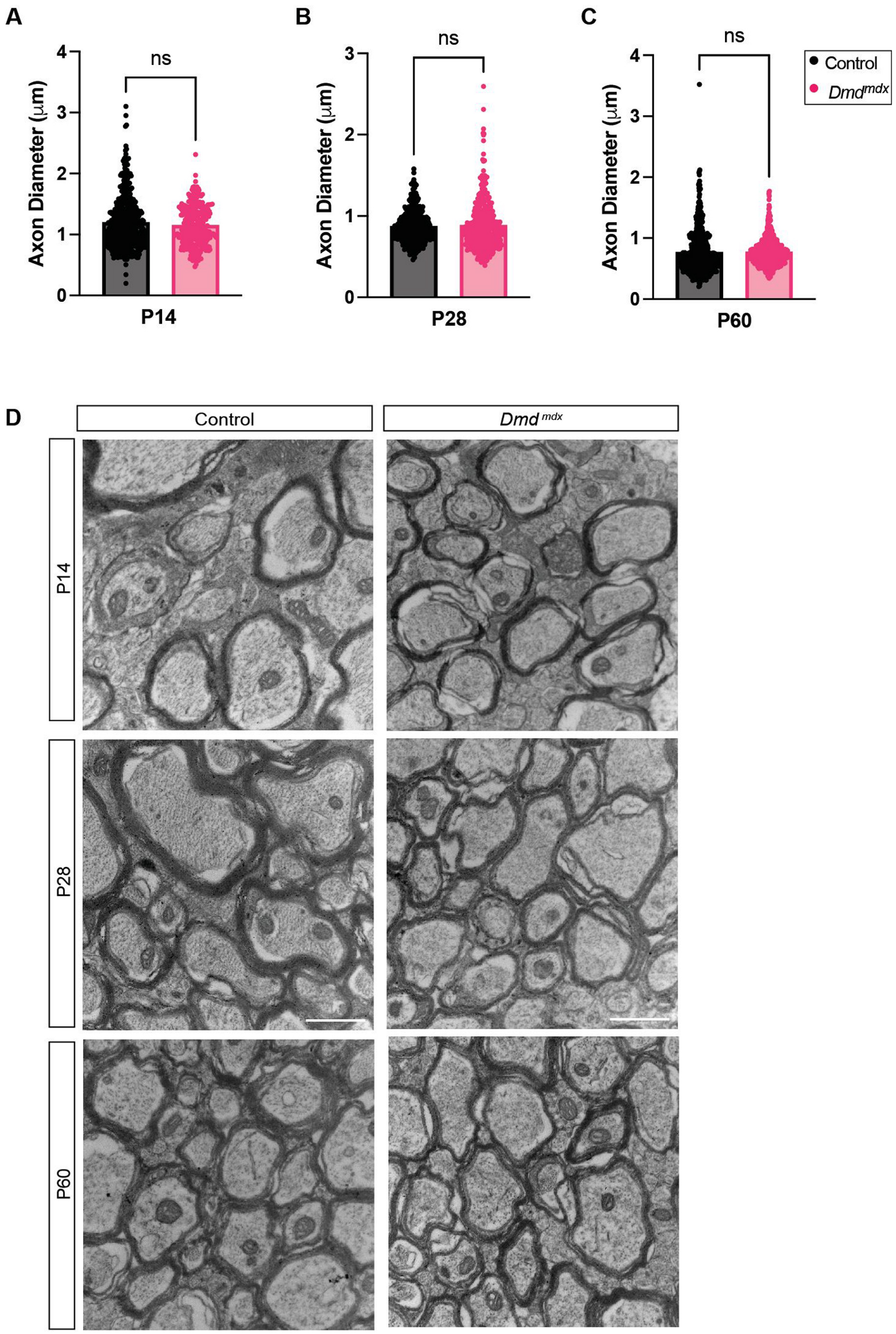

Fig. S7. Axon diameters in control and *Dmd*^{mdx} myelinated axons.

Diameters of myelinated axons at P14 (A), P28 (B), and P60 (C) in control and *Dmd*^{mdx} mice. A minimum of 274 axons were measured per condition. Unpaired two-tailed t=test was performed. ns*, not significant.* (D) Higher magnification views of panels depicted in Figure 5. Scale bar, 1μm.

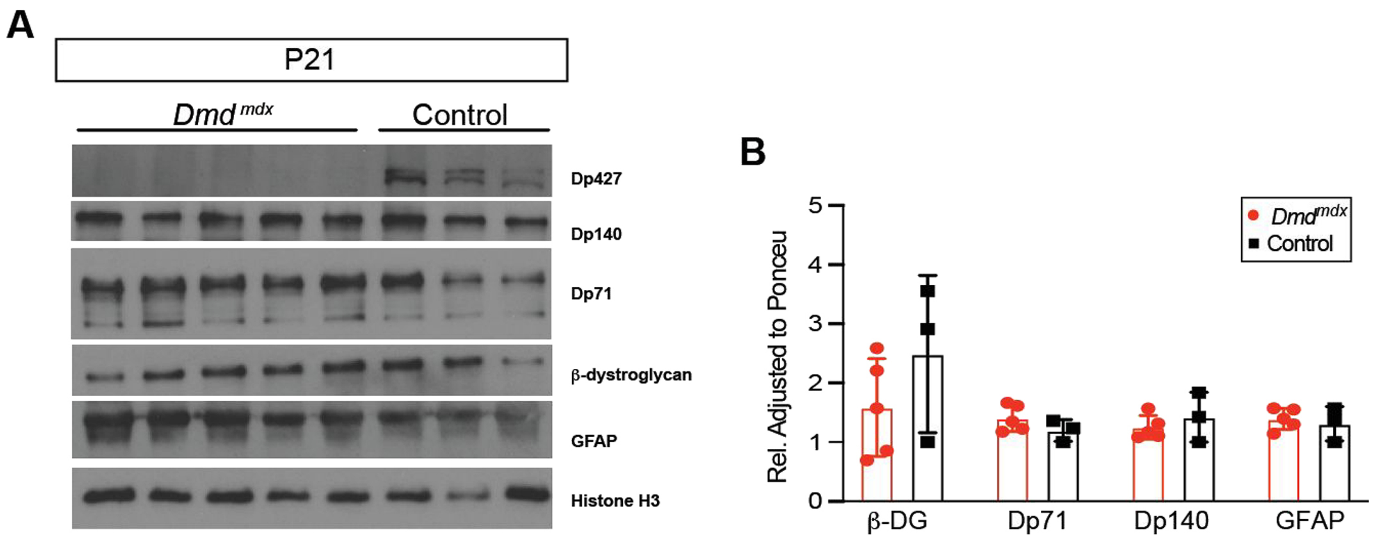

Fig. S8. Western blots to detect dystrophin, dystroglycan, and GFAP levels in the corpus callosum of P21 *Dmd^{mdx}* mice.

(A) Representative western blots of corpus callosum lysates from P21. Upper membranes were probed for dystrophin proteins (Dp) (427, 140, and 71 kDa). Other membranes were probed for β-dystroglycan (43 kDa), GFAP (50 kDa), and Histone H3 (15 kDa). (B) Quantification of western blot analysis. Unpaired two-tailed t-test was performed, with no significant differences. n=3 control and n=5 *Dmd^{mdx}*. Error bars denote SEM.
